# Development of high-energy non-aqueous lithium-sulfur batteries via redox-active interlayer strategy

**DOI:** 10.1038/s41467-022-31943-8

**Published:** 2022-08-08

**Authors:** Byong-June Lee, Chen Zhao, Jeong-Hoon Yu, Tong-Hyun Kang, Hyean-Yeol Park, Joonhee Kang, Yongju Jung, Xiang Liu, Tianyi Li, Wenqian Xu, Xiao-Bing Zuo, Gui-Liang Xu, Khalil Amine, Jong-Sung Yu

**Affiliations:** 1 Department of Energy Science and Engineering, Daegu Gyeongbuk Institute of Science & Technology (DGIST), Daegu, 42988 Republic of Korea; 2 Chemical Sciences and Engineering Division, Argonne National Laboratory, 9700S Cass Ave, Lemont, IL 60439 US; 3 Department of Nanoenergy Engineering, Pusan National University, Busan, 46241 Republic of Korea; 4 Department of Chemical Engineering, Korea University of Technology and Education (KOREATECH), Cheonan, 330-708 Republic of Korea; 5 X-ray Science Division, Argonne National Laboratory, 9700S Cass Ave, Lemont, IL 60439 US; 6 Materials Science and Engineering, Stanford University, Stanford, CA USA; 7 Materials Science, Energy and Nano-engineering Department, Mohammed VI Polytechnic University (UM6P), Ben Guerir, Morocco; 8 Energy Science and Engineering Research Center, DGIST, Daegu, 42988 Republic of Korea

**Keywords:** Materials for energy and catalysis, Batteries, Energy storage, Materials science

## Abstract

Lithium-sulfur batteries have theoretical specific energy higher than state-of-the-art lithium-ion batteries. However, from a practical perspective, these batteries exhibit poor cycle life and low energy content owing to the polysulfides shuttling during cycling. To tackle these issues, researchers proposed the use of redox-inactive protective layers between the sulfur-containing cathode and lithium metal anode. However, these interlayers provide additional weight to the cell, thus, decreasing the practical specific energy. Here, we report the development and testing of redox-active interlayers consisting of sulfur-impregnated polar ordered mesoporous silica. Differently from redox-inactive interlayers, these redox-active interlayers enable the electrochemical reactivation of the soluble polysulfides, protect the lithium metal electrode from detrimental reactions via silica-polysulfide polar-polar interactions and increase the cell capacity. Indeed, when tested in a non-aqueous Li-S coin cell configuration, the use of the interlayer enables an initial discharge capacity of about 8.5 mAh cm^−2^ (for a total sulfur mass loading of 10 mg cm^−2^) and a discharge capacity retention of about 64 % after 700 cycles at 335 mA g^−1^ and 25 °C.

## Abstract (web summary)

Lithium-sulfur batteries promise high energy density, but polysulfide shuttling acts as a major stumbling block toward practical development. Here, a redox-active interlayer is proposed to confine polysulfides, increase the cell capacity and improve cell cycle life.

## Introduction

The ever-growing demand for high-energy and low-cost batteries has boosted the exploration of novel electrochemistry beyond conventional lithium-ion batteries. Li-S batteries have drawn much attention because of their high theoretical energy density (2600 Wh kg^−1^) and the earth abundance of sulfur resources^1–6^. A typical Li-S battery is composed of a Li metal anode and a sulfur cathode, separated by a polypropylene separator and a Li^+^-conducting organic electrolyte^4,7^. The commercialization of Li-S batteries has been hindered by their low practical energy density, poor cycle life, and severe self-discharg^8–10^. These drawbacks are directly related to the dissolution/shuttling of long-chain lithium polysulfides (LiPSs) during battery operation^1–4^. A conventional strategy to mitigate these problems is to immobilize LiPSs within a rational sulfur host via physical/chemical adsorption, such as conductive carbon^11–14^, polar materials^15–17^, or single-atom catalyst^18–20^. These approaches have resulted in considerable improvement on the charge-discharge cycling stability of the Li-S battery. However, these improvements were obtained at the expense of relatively lower areal sulfur loading and the excess amount of electrolytes (see Supplementary Information Table 1). A grand challenge remains for the achievement of long cycle-life sulfur cathodes with high areal capacity (≥6 mAh cm^−2^) and lean electrolyte condition (<5 µl mg^−1^) to enable high practical energy density for Li-S batteries^21,22^.

Another prominent strategy to enable a durable Li-S battery is to restrain the dissolved LiPSs in the cathode side and therefore prevent the associated active material loss and Li metal corrosion. Indeed, the LiPSs formed at the cathode generate a concentration gradient which accelerates the diffusion of LiPSs and, thus, the reaction with the lithium metal electrode by causing detrimental surface reactions^23–25^. This has triggered much effort in the development of a multifunctional conducting interlayer (IL), including carbonaceous-^26–30^, polymer-^31,32^, and metal based^33,34^. This interlayer serves as an electrical conductive extension zone and also a barrier layer between cathode and separator to reuse and immobilize LiPSs in order to improve cycle stability. The opposite approach, developing electrical insulating but polar ILs, has been generally considered as significantly less effective because they cannot provide the necessary electronic transport for sulfur and would lead to low sulfur utilization and large voltage polarization as well as permanent capacity loss^35^. Use of an ion-selective separator is another promising way to block LiPSs shuttling, which induces extra resistance to transport of lithium ions and thus exhibits voltage polarization under fast charge^36^. Nevertheless, all these strategies have limited practical application because of their low areal capacity loading (<5 mAh cm^−2^) in the validated cathodes (see Supplementary Information Tables 2 and 3), which significantly limits the practical energy density of Li-S batteries.

Another critical problem that has been ignored is the trade-off between cycle life and practical energy density of Li-S batteries due to the addition of ILs. This occurs because ILs are redox-inactive in the tested voltage range of Li-S batteries. Therefore, despite the gain in the cycle stability, the thick and non-active ILs will cause considerable weight increase for practical cells, leading to notable reduction on the practical specific energy and energy density^23^. The development of thin and lightweight ILs is crucial but can only mitigate this problem to some extent^33,37^.

Here, we present a transformative approach to boost the practical energy density and cycle life of Li-S batteries by using redox-active ILs. We use sulfur-impregnated polar ordered mesoporous silica composites as the ILs for Li-S batteries. The insulating S-embedding IL can accommodate high areal S loading (≥14 mg cm^−2^) and enable a higher areal capacity (>10 mAh cm^−2^). Moreover, the optimized IL shows good cycle stability with high areal capacity for over 500 cycles (3.85 mAh cm^−2^) under high specific current (1675 mA g^−1^) and low electrolyte/sulfur (E/S) ratio (10 µl mg^−1^).

## Results

### Design rationale of redox-active ILs

**Fig. 1:**
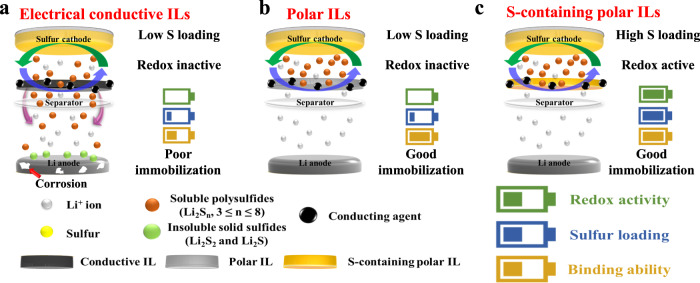
Design rationale of redox-active ILs. **a**–**c**, Illustration of Li-S batteries with (**a**) electrical conductive, (**b**) polar, and (**c**) S-containing polar interlayers (ILs).

Figure 1 shows the design rationale of the developed redox-active ILs versus conventional electrical conductive and polar ILs for Li-S batteries. The conductive carbon-based ILs suffer from low areal S loading, redox inactivity, and insufficient LiPSs immobilization due to the poor interaction of nonpolar carbon with polar LiPSs, resulting in limited cycle life and low practical energy density (Fig. 1a and Supplementary Information Table 2). Due to strong polar-polar interaction, the polar ILs exhibit better confinement towards LiPSs, but remain electrochemically inactive and limited in areal S loading (Fig. 1b and Supplementary Information Table 3). Having noticed that sulfur has the highest specific capacity in the tested voltage range, we designed and synthesized redox-active ILs (Fig. 1c) consisting of sulfur-impregnated composites of polar platelet ordered mesoporous silica (denoted as pOMS/S_x_, where x% represents the sulfur weight ratio in the composites).

The developed redox-active IL design is based on the following rationale. First, the pOMS/S_x_ itself can provide additional active sulfur and boost the areal capacity of the cell, in contrast with the widely reported redox-inactive ILs that has added extra weight of inactive materials into the cell despite improved cycling stability. This behaviour is different from the previously reported thin interlayer materials such as Mo_6_S_8_^38^. Although it can be considered as electrochemically active, the Mo_6_S_8_ interlayer contributes little capacity (100–150 mAh g^−1^) and can only enable a low areal capacity of 3 mAh cm^−2^. Moreover, despite high inherent sulfur content, the polar pOMS/S_x_ ILs can still effectively immobilize the dissolved LiPSs on the surface or within the porous structures via the polar-polar interactions, and thus can hinder the shuttle effect of LiPSs and Li metal corrosion, leading to improved cycling stability (Supplementary Fig. 1a). In particular, the separated polysulfides trapping sites (polar silica) and electron transfer sites (electron-conducting agent) can maximize the reaction efficiency of Li-S cells with pOMS/S_x_ ILs during charge-discharge. In addition, the platelet morphology can accommodate the volume changes of sulfur that occur during repeated lithiation/de-lithiation. Although pOMS/S_x_ could be used as cathode directly as demonstrated in previous research works^39^, we do find further beneficial effects in enabling higher areal capacity loading cells when pOMS/S_x_ is used as interlayer. The redox-active interlayer could provide additional sulfur to achieve high cell sulfur loading, which can alleviate the challenge (e.g., laminate cracking, electron transport) on the high S loading cathodes. With this interlayer design, we attained high areal capacity (>10 mAh cm^−2^) with high sulfur loading (>10 mg cm^−2^) and good cycle stability (~700 cycles) for Li-S batteries (Supplementary Fig. 1b).

### Structures of ILs and the interaction with polysulfides

**Fig. 2:**
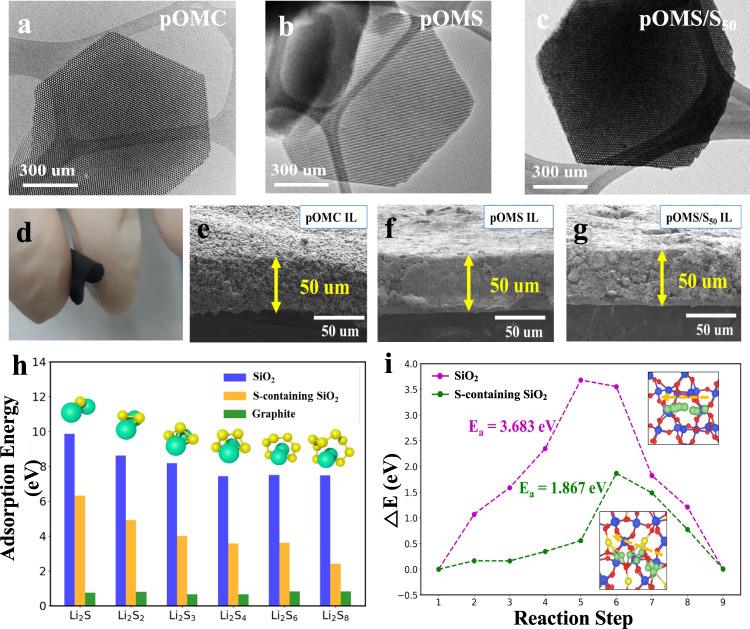
Physicochemical and theoretical characterizations of the interlayers. **a**–**c** TEM images of (**a**) pOMC, (**b**) pOMS, and (**c**) pOMS/S_50_ composite. **d** Digital photograph of pOMS IL showing its flexibility. **e**–**g** Cross-sectional SEM images of (**e**) pOMC, (**f**) pOMS, and (**g**) pOMS/S_50_ ILs. **h** Calculated adsorption energies of Li_2_S_x_ (x = 1, 2, 3, 4, 6, and 8) on SiO_2_ (blue), S-containing SiO_2_ (orange), and graphite (green). **i** Potential energy profiles of Li-ion diffusion along different reaction steps on the surface of the SiO_2_ and S-containing SiO_2_. (Inset is minimum energy path for Li-ion diffusion on SiO_2_ and S-containing SiO_2_).

We prepared three redox-active pOMS/S_x_ ILs with sulfur content of 30%, 50%, and 70% (see thermogravimetric analysis in Supplementary Fig. 2). To further demonstrate the effectiveness and advantages of our redox-active IL concept, we prepared another two parallel ILs with similar morphologies for apple-to-apple comparison: platelet ordered mesoporous silica (denoted as pOMS) and platelet ordered mesoporous carbon (denoted as pOMC, prepared from template replication of the pOMS). All the as-prepared materials (Supplementary Fig. 3) exhibit a hexagonal platelet-shaped nanostructure with similar mesopore channel length (~200 nm) and plate diameter (~1 μm). Hexagonally ordered mesopore channels with a uniform mesopore size (~7 nm for pOMS and ~4 nm for pOMC) can be appreciated in transmission electron microscopy (TEM) images for pOMC and pOMS (Fig. 2a and b). After sulfur infiltration, part of the mesopore channels was filled with sulfur (Fig. 2c). High-angle annular dark-field scanning transmission electron microscopy (HAADF-STEM) and element mapping show that the sulfur is homogeneously distributed throughout the pOMS/S_50_ (Supplementary Fig. 4). The infiltration of sulfur was further confirmed by small-angle X-ray diffraction (SAXRD) (Supplementary Fig. 5), wide-angle X-ray diffraction (WAXRD) (Supplementary Fig. 6), and N_2_ isothermal adsorption-desorption measurements (Supplementary Fig. 7 and Supplementary Information Table 4).

All the as-prepared powder materials were then mixed with Ketjenblack as a conducting agent and polytetrafluoroethylene binder in isopropyl alcohol to fabricate free-standing, flexible films (Fig. 2d and Supplementary Fig. 8). Cross-section scanning electron microscopy (SEM) images confirm that all the ILs have a uniform thickness of approximately 50 μm (Fig. 2e–g). Moreover, contact angle tests show that pOMS and pOMS/S_50_ demonstrate better electrolyte wettability than pOMC IL (Supplementary Fig. 9), indicating that polar pOMS and pOMS/S_50_ ILs can facilitate the electrolyte penetration and improve the transport of lithium ions. The pore structure of various ILs was further confirmed by mercury intrusion measurements (Supplementary Fig. 10), and the detailed surface porosity and total intrusion volume for pOMS, pOMC, and different pOMS/S ILs are summarized in Supplementary Information Table 5.

First-principles density functional theory (DFT) calculation was performed to understand the interaction between LiPSs and different ILs. The adsorption energies of Li_2_S_n_ (*n* = 1–4, 6, and 8) on the (001) surface of graphite, SiO_2_, and sulfur-containing SiO_2_ were calculated (Supplementary Figs. 11–13). The results show that the lithium atoms in the Li_2_S_n_ species can bond with the oxygen atoms on the surface of polar SiO_2_ or S-containing SiO_2_, while the sulfur atoms (Li_2_S_n_) can interact with silicon atoms. Thus, the adsorption energies of Li_2_S_n_ species on the SiO_2_ and S-containing SiO_2_ surface are thus both much higher than those on the graphite system (Fig. 2h) and are in the order SiO_2_ > S-containing SiO_2_ » graphite. Therefore, both pOMS and pOMS/S_x_ IL could effectively confine dissolved LiPSs during charge/discharge cycling. As previously indicated by Cui and co-workers^16^, it is also important to balance surface adsorption and diffusion of LiPSs, especially on non-conductive oxides. Lithium-ion diffusion calculation (Fig. 2i) shows that the S-containing SiO_2_ exhibits a significantly lower Li^+^ diffusion barrier than bare SiO_2_ and can thus favour the electrochemical reaction kinetics of LiPSs during charge and discharge.

**Fig. 3:**
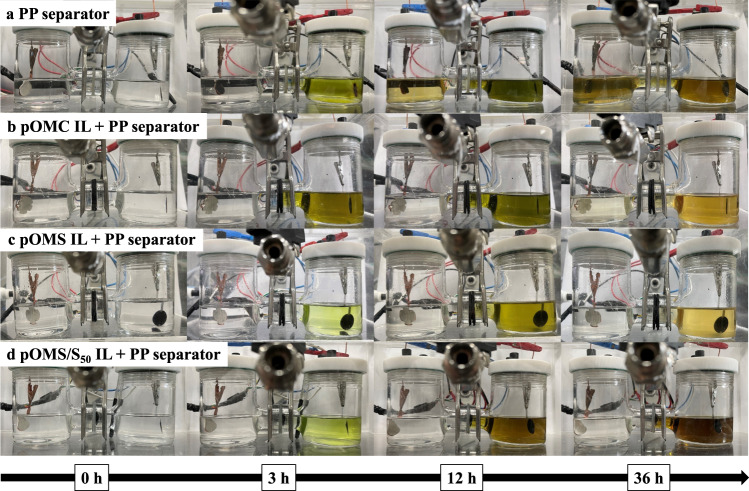
Lithium polysulfides diffusion tests using interlayers. Digital photographs of visual electrochemical tests performed in H-type cells at 167.5 mA g^−1^ for 36 h using the PP (polypropylene) separator film with (**a**) IL-free, (**b**) pOMC IL, (**c**) pOMS IL, and (**d**) pOMS/S_50_ IL (Sulfur cathode was placed on the right-hand side and Li metal on the left-hand side of the H-type cell).

To investigate the restraining effect of the IL on the shuttle of LiPSs, the electrochemical investigations were carried out through the H-type cells separated by the PP separator film with IL-free, pOMC IL, pOMS IL, and pOMS/S_50_ IL to observe the LiPSs diffusion process during the real-time electrochemical reaction (Fig. 3). For the IL-free cell, the colour of the anode chamber gradually changes from white to brown as the discharging time increased (Fig. 3a). Moreover, the pOMC IL cell turned into a bright yellow solution in the anode chamber after 36 h (Fig. 3b), which cannot completely prevent polysulfides diffusion. However, pOMS (Fig. 3c) and pOMS/S_50_ (Fig. 3d) ILs were transparent in the anode chamber even after 36 h. In addition, for the pOMS/S_50_ IL cell, the cathode chamber shows a dark brown colour probably due to the higher sulfur loading. Therefore, pOMS and pOMS/S ILs were effective in preventing the LiPSs shuttling phenomenon.

To further probe the interaction between LiPSs and different ILs, we also conducted visual LiPSs diffusion tests using an H-type cell (Supplementary Fig. 14a) with 0.1 M Li_2_S_6_ in dimethoxyethane/dioxolane (DME/DOL) solution in the left chamber and pure DME/DOL solvent in the right chamber. The LiPSs solution in the left chamber would spontaneously diffuse into the right chamber through the IL because of the concentration gradient between the two chambers, while the IL could serve as a shielding layer to prevent the migration of LiPSs. For pOMC IL (top panel), LiPSs were present in the right chamber after 24 h, and the colour of the right chamber got darker gradually and turned brown after 36 h, indicating that pOMC IL fails to completely prevent the diffusion of LiPSs over an extended period. By sharp contrast, in the case of pOMS IL (middle panel) and pOMS/S_50_ IL (bottom panel), the right chamber still maintained a clear and transparent solution even after 36 h, which can be attributed to their effective adsorption for LiPSs.

UV-visible absorption spectra were further carried out to measure the Li_2_S_6_ concentration in the right chamber solution after H-cell testing for 36 h (Supplementary Fig. 14b). As shown, the starting Li_2_S_6_ solution shows a broad peak in the wavelength range of 250–300 cm^−1^ due to the presence of S_6_^2−^ species. After H-cell testing, the solutions in the pOMS and pOMS/S_50_ IL cells show a weaker absorbance intensity compared to that in the pOMC IL, confirming that both polar pOMS and pOMS/S_50_ ILs are a reliable barrier to prevent LiPSs migration. SAXRD further indicates the existence of LiPSs in the soaked pOMS, leading to a notable intensity decrease, while the characteristic peaks of pOMC with its 2D hexagonal p6mm pore structure are still well maintained (Supplementary Fig. 14c). X-ray photoelectron spectroscopy (XPS) also reveals that the binding energy of Si 2p of pOMS and pOMS/S_50_ shifted to a lower value after the interaction with Li_2_S_6_ (Supplementary Fig. 14d). Similar results can be seen in the S 2p XPS spectra of soaked pOMS and pOMS/S_50_ ILs (Supplementary Fig. 15). The results confirm the interaction between the negative S_n_^2−^ anions and positive Si sites, demonstrating that polar pOMS and pOMS/S_50_ IL can provide efficient anchoring sites for LiPSs during charge and discharge.

### Electrochemical energy storage characterizations

The cells that are IL-free or have pOMC IL, pOMS IL, and pOMS/S_x_ ILs were tested with a bare sulfur electrode (70 wt.% of sublimed sulfur in the electrode) having an areal S loading of 2.5 mg cm^−2^. All the cells were run at a charge/discharge specific current of 167.5 mA g^−1^ for the first 4 cycles, and then were charged and discharged at the desired specific current. The total cell sulfur loadings and electrochemical conditions of all the tested cells are summarized in Supplementary Information Table 6. The specific capacities, areal capacities, charge/discharge specific current and electrolytes/sulfur ratio are all based on the mass of total S loading in the cells (include both the cathode and the interlayer).

To evaluate the optimal thickness of the IL, the pOMS ILs were made in varying thicknesses of 30, 50, and 80 μm (Supplementary Fig. 16). Among three ILs, the pOMS IL with 50 μm exhibits the best cycling performances due to the improved LiPSs adsorption and Li-ion diffusion. Therefore, the current work focused on the pOMS IL with 50 μm.

Cyclic voltammetry shows that the pOMS IL cell exhibits no visible decay and more reversible redox reactions than the IL-free and pOMC IL cells (Supplementary Fig. 17).

**Fig. 4:**
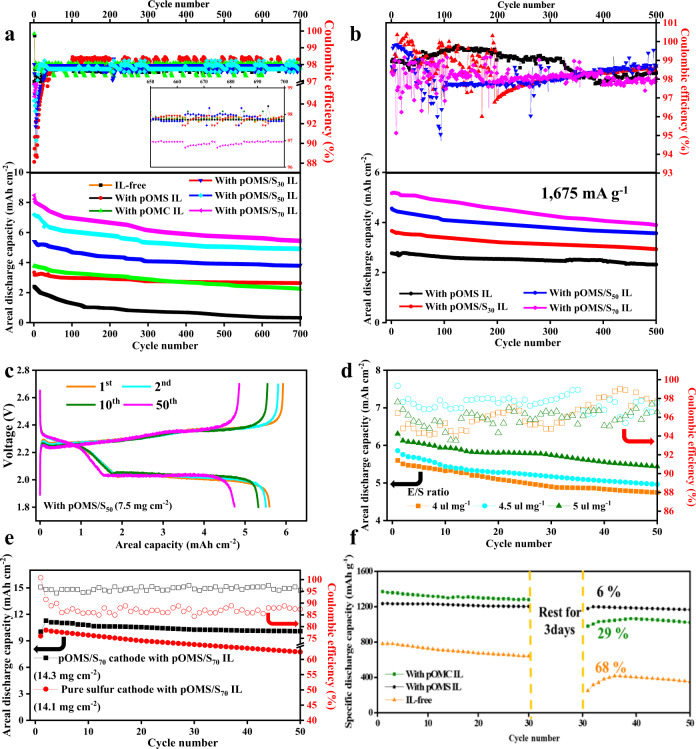
Electrochemical energy storage performance of Li-S cells with and without interlayers. **a** Cycling performances of IL-free and different IL cells at a charge/discharge specific current of 335 mA g^−1^ for 700 cycles at 25 °C. **b** High specific current cycle performance with pOMS IL and pOMS/S_x_ ILs at 1675 mA g^−1^ for 500 cycles at 25 °C. The top and bottom panels in a and b represent the Coulombic efficiency and areal discharge capacity verse cycle number, respectively. **c** Galvanostatic charge-discharge profiles under E/S ratio of 4 µl mg^−1^ at 25 °C. **d** Cycle performances of pure sulfur cathode with pOMS/S_50_ IL under different E/S ratios at 167.5 mA g^−1^ for 50 cycles at 25 °C. Hollow and solid symbols represent coulombic efficiency and areal discharge capacity, respectively. **e** Cycling performance of pure sulfur cathode (The pure sulfur cathode was composed of sublimed sulfur 70 wt. %, binder 10 wt. %, and electron-conducting agent 20 wt. %) with pOMS/S_70_ IL (total sulfur loading of 14.1 mg cm^−2^, see Supplementary Information Table 6) and pOMS/S_70_ cathode with pOMS/S_70_ IL (total sulfur loading of 14.3 mg cm^−2^) under E/S ratio of 5 µl mg^−1^ at 167.5 mA g^−1^ for 50 cycles. **f** Self-discharge behaviour of different cells at 502.5 mA g^−1^ at 25 °C after rest time of 3 days at room temperature. Total sulfur content includes both the cathode and the interlayer.

The cycling performances of the cells with different ILs with an E/S ratio of 10 µl mg^−1^ were then measured at 335 mA g^−1^ for 700 cycles (Fig. 4a). Because of the higher electronic conductivity of pOMC than pOMS, the pOMC IL cell has a higher initial discharged areal capacity of 3.8 mAh cm^−2^ than the IL-free (2.4 mAh cm^−2^) and pOMS IL (3.3 mAh cm^−2^) cells. After 700 cycles, the IL-free, pOMC IL, and pOMS IL cells delivered an areal capacity of 0.3, 2.2, and 2.6 mAh cm^−2^, corresponding to a capacity retention of 13, 58, and 79 %, respectively. The improved capacity retention of the pOMS IL cell clearly indicates a minor loss of active sulfur in the electrolytes during long-term cycling, which should be due to the strong polar-polar interaction between pOMS IL and LiPSs. Furthermore, the pOMS and pOMS/S_x_ ILs also enable long-term performance with high areal capacity and minimal voltage polarization (Supplementary Fig. 18).

Interestingly, despite their poor electronic conductivity, the pOMS/S_x_ ILs significantly increased the overall areal capacity of the cells without compromising the cycle stability. As shown in Fig. 4a, the cells with pOMS/S_30_, pOMS/S_50_, and pOMS/S_70_ ILs exhibited areal discharge capacities of 5.4, 7.2, and 8.5 mAh cm^−2^, respectively. After 700 cycles, the reversible areal discharged capacities were still maintained at 3.8, 4.9, and 5.4 mAh cm^−2^, corresponding to a capacity retention of 70, 68 and 64 %, respectively. Ex situ high energy X-ray diffraction measurements of cycled interlayers revealed that the S-containing ILs could not only trap polysulfides, but also contribute to the redox reaction of embedded sulfur and trapped polysulfides during charge/discharge (Supplementary Fig. 19). Moreover, the curves converted from areal capacity to specific and volumetric capacities show the same cycle stability behaviour (Supplementary Figs. 20 and 21). The pOMS/S_70_ IL cell (7.5 mg cm^−2^) has a high sulfur loading compared to pOMS/S_50_ IL cell (5 mg cm^−2^). Thus, pOMS/S_70_ IL cell has high areal capacity than that of the cell with pOMS/S_50_ IL. However, the current applied to the charge/discharge processes increases with increasing sulfur loading at a constant specific current. Thus, the current increase during electrochemical energy storage testing could affect the stripping/plating reversibility of Li metal anode^40–42^. Meanwhile, the pOMS/S_70_ IL contains higher sulfur content than pOMS/S_50_ IL, leading to decreased polysulfide confinement effectiveness. Thus, although the cell with pOMS/S_70_ IL has a higher areal capacity than that of pOMS/S_50_ IL, the latter shows better cycle stability. The long-term cycling performances of the cells with pOMS and pOMS/S_x_ ILs were further evaluated at a higher specific current of 1,675 mA g^−1^ (Fig. 4b). As shown, the pOMS, pOMS/S_30_, pOMS/S_50_, and pOMS/S_70_ ILs cells still retain a high areal capacity of 2.32, 2.92, 3.57, and 3.85 mAh cm^−2^ after 500 cycles, corresponding to a low capacity decay rate of 0.03, 0.04, 0.043, and 0.051 % per cycle, respectively. Additionally, pOMS/S_70_ IL cell was tested at a higher charge/discharge specific current of 3350 mA g^−1^ for 150 cycles (Supplementary Fig. 22). The result indicating that sulfur-containing pOMS IL can mitigate the polysulfides migration despite high sulfur loading in the IL and high specific current applied. These results demonstrate the advantages of our redox-active IL concept in constraining LiPSs and boosting cell areal capacity. These advantages have been confirmed by a comprehensive comparison with previously reported redox-inactive ILs in terms of cycle life, capacity retention, and areal capacity (Supplementary Fig. 23 and Supplementary Information Tables 1–3, and 7).

We further evaluated the electrochemical energy storage performance of the cells at lower E/S ratio to enable higher practical specific energy. As shown in Fig. 4c and d, even with an E/S ratio of 4–5 µl mg^−1^, the cells with pOMS/S_50_ IL still demonstrated high areal capacity (~5–6 mAh cm^−2^) and excellent cycle life as well as stable voltage profiles at 167.5 mA g^−1^. Moreover, combining redox-active ILs with an encapsulated sulfur cathode achieved a comparable areal mass loading (~10 mg cm^−2^) with conventional intercalation cathodes^5^. The test results with pOMS/S_70_ IL showed a high areal capacity of over 10 mAh cm^−2^ with stable cycle life at an E/S ratio of 5 µl mg^−1^ (Fig. 4e). The projected cell-specific energy is ~285.7 Wh kg^−1^ according to the calculated plot of specific energy verse areal S loading and E/S ratio in Supplementary Fig. 24^9^.

In addition to cycle life, Li-S batteries suffer from self-discharge and reduced shelf life due to the reactivity of LiPSs^10^, which hinder their commercialization but has been paid less attention. Figure 4f shows the self-discharge behaviours in various coin cells using different ILs after resting for 3 days at room temperature. As shown, the IL-free and pOMC IL cells undergo a capacity loss of 68 and 29 %, respectively. By sharp contrast, the capacity loss of the pOMS IL cell is reduced to 6 %. Moreover, even with a longer resting period of 14 days at 45 ± 2 °C, the pOMS IL cell still exhibited a lower self-discharge rate than the IL-free and pOMC IL coin cells (Supplementary Fig. 25).

**Fig. 5:**
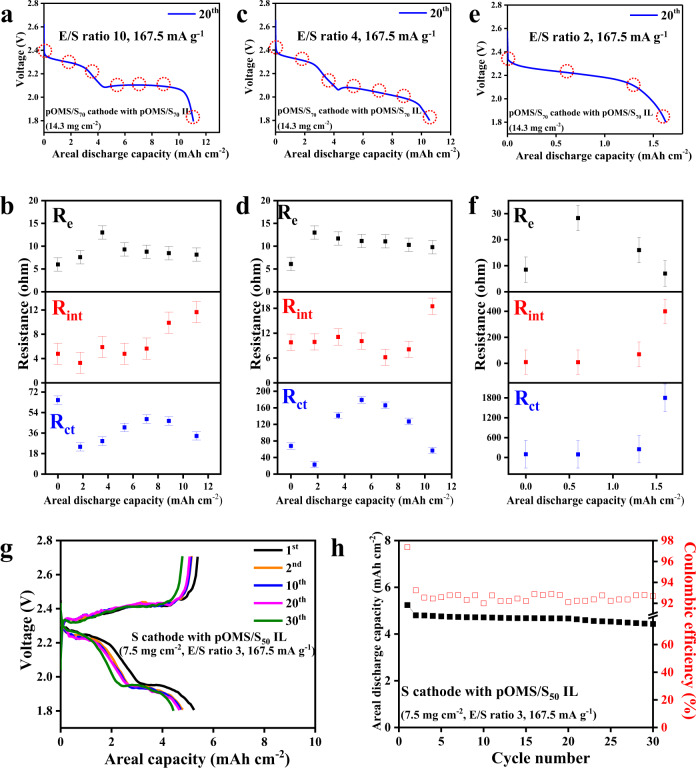
Electrochemical energy storage performance of Li-S cells at various E/S ratios. The discharge voltage curves and ex situ EIS results of the pOMS/S_70_ cathode with pOMS/S_70_ IL at 167.5 mA g^−1^ and E/S ratios of (**a**,**b**) 10 μL mg^−1^, (**c**,**d**) 4 μL mg^−1^, and (**e**, **f**) 2 μL mg^−1^. The red dotted circles correspond to the ex-situ point shown in Fig. 5d–f. The displayed error bar is the standard deviation value. (**g**) Charge/discharge voltage profiles and (**h**) cycling performance of sulfur cathode with pOMS/S_50_ IL at an E/S ratio of 3 μL mg^−1^.

It is well known that further reduction of E/S ratio (E/S ≤ 2 µl mg^−1^) is essential to achieve a practical specific energy of ≥400 Wh kg^−1^ at the cell level because of the weight percentage of electrolytes in the whole cell, which; however, remains one of the critical challenges for high-energy and durable Li-S cells^9^. In Li-S battery, the Li-ion transfer changes depend on the amount of electrolytes^22^. In general, the lower the electrolyte amount in the cell, the slower the Li-ion transfer^43^. To understand the behaviour of our cells under lean electrolytes operation, analyses of the discharge voltage profile and electrochemical impedance (EIS) measurements of the pOMS/S_70_ cathode with pOMS/S_70_ IL were carried out for different E/S ratios as shown in Supplementary Fig. 26 and Supplementary Information Table 8. As shown, for the cell with E/S ratio of 10 µl mg^−1^ (Fig. 5a and b) and 4 µl mg^−1^ (Fig. 5c and d), the cell can still exhibit two distinct discharge plateaus with interfacial and charge-transfer resistances below 20 Ω and 200 Ω, respectively. Therefore, the cells under E/S ratio of ≥4 µl mg^−1^ exhibit high S utilization and good cycling stability. In contrast, when the E/S ratio is reduced to 2 µl mg^−1^ (Fig. 5e), it shows only a short discharge plateau and increased internal resistance of the 400 Ω (Fig. 5f), which is mainly likely due to the substantial Li ion transfer constraint at the interface^43^.

Indeed, it has been well discussed in Li-S community that Li metal failure and electrolytes deterioration will play a dominant role in the performance of Li-S batteries under high-loading cathode and lean electrolytes condition^44–46^ On the one hand, it has been well known that the conventional DME/DOL electrolytes cannot enable efficient reversible Li stripping/plating^47^. For example, under a high S loading of 14.3 mg cm^−2^ and 167.5 mA g^−1^, the absolute specific current applied onto Li metal is as high as 2.4 mA cm^−2^, raising critical challenge (e.g., dendrite formation) for the stabilization of Li metal^22^. On the other hand, lithium metal is highly reactive in contact with non-aqueous fluorinated electrolyte solutions, which will inevitably consume considerable amount of electrolytes to form solid-electrolyte interphase (SEI). Under lean electrolytes condition, the continuous parasitic reactions between Li metal and electrolytes could result in electrolytes depletion of the cell, leading to large increase of the internal cell resistance (as shown in Fig. 5c) and hence sluggish reaction kinetics of sulfur cathodes.

The electrochemical test results in Fig. 5g and h show that the lowest E/S ratio we can achieve in coin cell is 3 µl mg^−1^, which can maintain a reversible areal discharge capacity of >4 mAh cm^−2^ within 30 cycles. We further fabricated a single-layer Li-S pouch cell using pure sulfur cathode (The pure sulfur cathode was composed of sublimed sulfur 70 wt. %, binder 10 wt. %, and electron-conducting agent 20 wt. %) and pOMS interlayer, which can exhibit the high specific capacity of ~1000 mAh g^−1^ in the first 5 cycle (Supplementary Fig. 27). However, the cell exhibits a rapid failure in the subsequent cycling, which is a characteristic of Li metal failure^21^. We believe that by integrating the redox-active interlayer concept with rational Li metal protection and optimization of electrolytes structures, it could unlock the operation of Li-S cells under very low E/S ratio.

### Ex situ postmortem characterizations of interlayers, separators and electrode

The cycled cells with different ILs were disassembled and characterized to investigate the stability of the lithium metal, separator, and ILs after cycling. As shown in Supplementary Fig. 28, both cycled lithium metal and separator in the pOMS and pOMS/S_50_ IL cells were well preserved in comparison with pristine Li metal and separator, while the IL-free and pOMC IL cells formed yellow deposits of LiPSs on the surface of the cycled separator and Li metal.

**Fig. 6:**
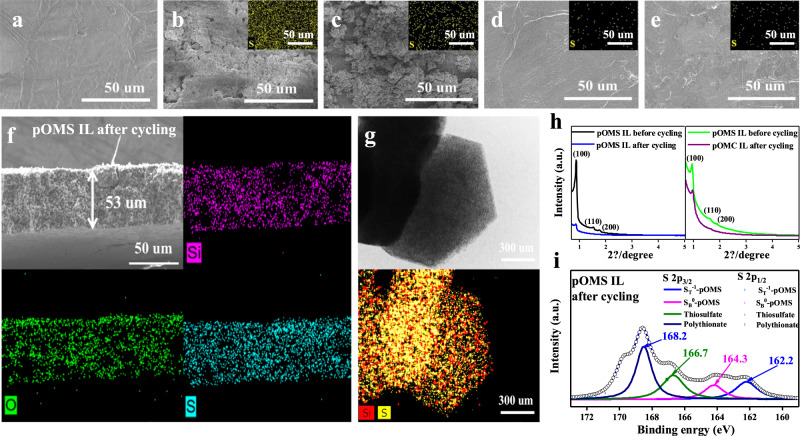
Ex situ postmortem characterizations of Li-S cells with and without interlayers. **a**–**e** Surface SEM images of (**a**) fresh Li anode and Li anodes from (**b**) IL-free, **(c)** pOMC IL, (**d**) pOMS IL, and (**e**) pOMS/S_50_ IL cells after 100 cycles at a charge/discharge specific current of 837.5 mA g^−1^ and the fully discharge state (Inset is the S mapping images of the Li surface). **f** Cross-sectional SEM image and EDS elemental mapping profiles of the pOMS IL after 500 cycles at 1,675 mA g^−1^ and the fully discharge state. **g** High-resolution TEM image and elemental mapping of pOMS IL after 100 cycles at 837.5 mA g^−1^ and 25 °C at the fully discharge state. **h**, SAXRD patterns of pOMS and pOMC IL before and after 500 cycles at 1,675 mA g^−1^ and 25 °C at the fully discharge state. **i** S 2p XPS spectrum of the pOMS IL obtained after 100 cycles at 837.5 mA g^−1^ and 25 °C at the fully discharge state.

SEM images of the Li metal anode were also obtained before and after cycling. The morphology of fresh Li metal is smooth and flat (Fig. 6a). However, the IL-free and pOMC IL show a very rough surface with significant S-containing deposits after cell cycling (Fig. 6b and c). Such a phenomenon can be attributed to the shuttling of LiPSs during the electrochemical reaction. By contrast, the cycled Li metal in the pOMS and pOMS/S_50_ ILs cells is a smoother with little sign of sulfur (Fig. 6d and e), demonstrating much more efficient inhibition of LiPSs shuttling. This led to a decreased internal cell resistance and charge transfer resistance for the pOMS and pOMS/S_50_ IL after cycling, as revealed by EIS measurement (Supplementary Fig. 29 and Supplementary Information Table 9).

The structures of the cycled pOMC and pOMS ILs were further characterized. Figure 6f shows the cross-section SEM image of cycled pOMS IL and the corresponding elemental mapping. The results show that the thickness of pOMS IL was increased from 50 to 53 μm, and an amount of LiPSs was trapped in the pOMS IL. In addition, TEM on the cycled pOMS IL shows that the platelet morphology can be still well maintained, and sulfur infiltration is evidenced during cycling (Fig. 6g). By sharp contrast, the sulfur signal in the cycled pOMC IL is weaker, indicating less LiPSs adsorption (Supplementary Figs. 30 and 31). Therefore, the cycled pOMS IL exhibited intensity decrease as revealed by SAXRD, while the peaks of cycled pOMC remained (Fig. 6h).

The S 2p XPS characterization on the cycled pOMS IL (Fig. 6i) reveals that the peaks for the terminal (162.2 eV) and bridge (164.3 eV) sulfur shifts to higher energy, compared to that for Li_2_S_6_ (terminal: 161.7 eV; bridge: 163.3 eV). This shift is attributed to the interaction between polar pOMS IL and polar LiPSs species. Furthermore, a broad signal of the thiosulfate (O_3_S-S, 166.7 eV) and polythionate (O_3_S-S_x_-S, 168.2 eV) complexes due to the reaction between LiPSs and oxygen atoms of the pOMS^39^ are also observed and confirm that LiPSs species are trapped by the pOMS IL during cycling. Thus, the pOMS IL effectively reduces the active material loss and extends the cycle life.

## Discussion

In summary, we propose a polar and redox-active interlayer concept for high-energy and long-cycling Li-S batteries, in which sulfur is embedded into a polar platelet ordered mesoporous silica to form an interlayer. Interestingly, sulfur storage/trapping occur at the polar silica while electron transfer at conducting agent in pOMS/S_x_ IL during charge-discharge. During the electrochemical processes, this interlayer not only fulfils the role of effectively preventing the shuttling of long-chain polysulfides, but also contributes to enhance the areal capacity to the cell. The cell with optimal interlayer delivers an areal capacity of >10 mAh cm^−2^ with the benefit of high sulfur loading of >10 mg cm^−2^ and stable cyclability for 700 cycles, even under high specific current cycling and low electrolyte/sulfur ratio. These attributes can increase the practical specific energy of Li-S batteries.

## Methods

### Synthesis of platelet ordered mesoporous silica (pOMS)

Typically, 4.0 g of Pluronic P123 triblock copolymer (Aldrich, Mn = 5,800 g mol^−1^) was added into 0.32 g of ZrOCl_2_ and 80 g of 2.0 M HCl solution, and stirred overnight at 35 °C. Then, 4.2 g of tetraethyl orthosilicate (TEOS, Aldrich, 98 %) was added, and the mixture was stirred at the same temperature for 2 h. Afterward, the reaction solution was kept for an additional 24 h at 90 °C in an oven without any stirring. The resulting product was filtered, washed repeatedly with deionized water, and dried at 25 °C. The as-synthesized pOMS was then calcined in air at 550 °C for 5 h to remove the organic template from the pOMS framework.

### Synthesis of platelet ordered mesoporous carbon (pOMC)

The pOMC was prepared through template replication of the pOMS. The preparation procedure is as follows. 1.0 g of dry pOMS was mixed with 0.35 g of phenol and heated at 100 °C for 12 h. The resulting phenol-incorporated OMS composite was reacted with 0.3 g of paraformaldehyde at 160 °C for 8 h to produce a phenol/paraformaldehyde resin-pOMS composite. The composite polymer was carbonized at 900 °C for 3 h under Ar flow, and then treated in HF solution (40 wt% deionized water) at room temperature for 12 h to selectively remove the silica. The silica-free pOMC replica was obtained by washing with deionized water and drying overnight at 80 °C.

### Preparation of pOMS/S composites

The pOMS/sulfur (pOMS/S_x_) composites were prepared by following a conventional melt-diffusion method. The calcined pOMS and sulfur with different pOMS:S weight ratios (7:3, 1:1, and 3:7, denoted as pOMS/S_30_, pOMS/S_50_, and pOMS/S_70_, respectively) were ground together, transferred into a Teflon bottle, and heated at 155 °C for 12 h to obtain the pOMS/S_x_ composites.

### Synthesis of pOMC, pOMS, and pOMS/S composite interlayers

Three types of films (pOMC or pOMS or pOMS/S_x_ composite) were used as an interlayer (IL) of the Li-S battery. The pOMC, pOMS, and pOMS/S_*x*_ composites were mixed with Ketjenblack and a polytetrafluoroethylene binder in isopropyl alcohol. The mixture was pressed using a roll-press machine (WCRP-1015G, Wellcos) with a target thickness of 50 um and dried under vacuum for 12 h at 50 °C. Note that the weight ratio of composite to Ketjenblack to binder within the interlayer was 8:1:1 regardless of the type of interlayer. The sulfur loading in the pOMS/S_30_, pOMS/S_50_, and pOMS/S_70_ interlayers was approximately 2, 5, and 7.5 mg cm^−2^, respectively. The total sulfur loading in the cells with various interlayers was summarized in Supplementary Information Table 6.

### Permeation measurements

A Li_2_S_6_ solution with a concentration of 0.1 M was prepared with stoichiometric amounts of sulfur and Li_2_S in a molar ratio of 1:5 in DME (Aldrich, anhydrous, 99.5 %)/DOL (Aldrich, anhydrous, 99.8 %) solvent (1:1 v/v) under stirring for 24 h at 60 °C in an Argon-filled glovebox (O_2_ < 0.5 ppm, H_2_O < 0.5 ppm), which formed a brown solution.

Visual examination of the polysulfide-trapping effect was performed in an H-type cell in an Argon-filled glovebox. The devices consist of two glass cells and were separated by the pOMC, pOMS, pOMS/S_50_ ILs, respectively. The left chamber was filled with 0.1 M Li_2_S_6_ in DME and DOL solution, and the right chamber was injected with pure DME/DOL solvent to the same level.

### Coin cell assembly and electrochemical measurements

CR2032 (Hohsen Corporation, Japan) type coin cells with a sulfur cathode, interlayer, Celgard 2400 separator, and lithium anode were assembled in an argon-filled glove box (H_2_O  <  0.1 ppm, O_2_  <  0.1 ppm). The lithium metal has a thickness of 0.25 mm and a diameter of 15.6 mm. The cell was filled with a pair of wave spring and spacer over the lithium anode to ensure good electrical and mechanical contact. The separator (Celgard 2400) has a diameter of 18 mm. For sulfur cathode fabrication, a slurry composed of sulfur powder (70 wt. %), a conducting agent (Ketjenblack, 20 wt. %), and a polyvinylidene fluoride (PVDF) binder (10 wt. %) dissolved in N-methyl-2-pyrrolidone (NMP) was thoroughly mixed using a planetary mixer (Thinky ARE0319) under air atmosphere for 4 h and then coated on a carbon-coated Al foil (Dongwon Systems, 20 um) using a casting coater (MTI Corporation, MSK-AFA-I), followed by evaporation of the NMP for 12 h at 50 °C. The sulfur loading in the cathode was approximately 2.5 mg cm^−2^. The sulfur cathode has thickness of 45 μm. However, for cathodes with higher sulfur loading of about 6.5–7.0 mg cm^−2^, the slurry ratio was composed of 60 wt. % active material, 20 wt. % conducting agent, and 20 wt. % binder.

The electrolyte for these tests was 1.0 M lithium bis(trifluoromethanesulfonyl)imide (LiTFSI) dissolved in DME/DOL in a 1:1 volumetric ratio with 0.2 M LiNO_3_ as an additive. Unless otherwise noted, the volume of electrolyte/sulfur added for each cell was approximately 10 µl mg^−1^. The electrolyte/sulfur (E/S) ratio of the cell was calculated based on all the sulfur loading in both the cathode and the interlayer. In addition, the specific capacities, areal capacities, charge/discharge specific current and electrolytes/sulfur ratio are all based on the mass of total S loading in the cells (include both the cathode and the interlayer).

The cells were charged/discharged within 1.8 −2.7 V vs. Li/Li^+^ for the initial 4 cycles at a constant specific current of 167.5 mA g^−1^. After that, the cycling tests were conducted at various specific currents corresponding to the sulfur loadings indicated in Supplementary Information Table 6. The specific current refers to the mass of sulfur in the cathode and interlayer. Cyclic voltammetry (CV) measurement was carried out in an electrochemical workstation (Biologic VSP-1). The charge/discharge was conducted in a BaSyTec multichannel battery test system. Electrochemical impedance spectroscopy (EIS) was recorded in potentiostatic mode in a frequency range of 1 kHz to 100 mHz with an amplitude of 10 mV. The number of points per decade was set to 6 in all frequency range. All the electrochemical analyses were measured at 25 ± 2 °C, unless specified otherwise.

### Self-discharge test procedures

To check the self-discharge test, the cells were initially cycled for 30 cycles at 502.5 mA g^−1^ and 25 °C, then rested for 3 or 14 days before cycling for another 20 cycles. Self-discharge was calculated based on the discharge capacity of (30^th^–31^th^)/30^th^ × 100 %.

### Assembly of Li-S pouch cell

A pouch cell was assembled in the Ar-filled glove box (H_2_O < 0.1 ppm, O_2_ < 0.1 ppm), and the S cathode with the areal loading of 2.5 mg cm^−2^ was cut to the size of 3 cm × 4 cm. An Al tap was riveted on the cathode, and a Ni tab (thickness of 0.09 mm, MTI corporation) was riveted on the Cu foil (thickness: 5 μm, sigma-Aldrich). A piece of 3 cm × 4 cm Li metal foil with the thickness of 200 µm (MTI corporation) was put on the surface of Cu foil. Then a layer of Celgard 2400 separator was put on the surface of Li foil, and the pOMS interlaye,r as well as the S cathode was subsequently put on the top of separator. After injecting 750 µL electrolyte, the package was sealed under vacuum to get the Li-S pouch cell.

### Materials characterization

The morphologies and structures of the synthesized samples were characterized by scanning electron microscopy (SEM, Hitachi S-4700) operated at 10 kV and by transmission electron microscopy (TEM, EM 912 Omega) operated at 120 kV. The elemental mappings were performed on a scanning transmission electron microscope (STEM) with a high-angle annular-dark-field (HAADF) detector (Hitachi HF-3300). The phase analyses of the synthesized samples were confirmed by small-angle X-ray diffraction (SAXRD, Empyrean diffractometer system in the scan range of 2θ = 0.6–5.0°) as well as wide-angle X-ray diffraction (WAXRD, Rigaku Smartlab diffractometer with Cu-Kα radiation operating at 40 kV and 20 mA). The high-energy X-ray diffraction patterns of cycled pOMS/S50 ILs at different charge/discharge states were conducted at 17-BM of Advanced Photon Source of Argonne National Laboratory with a wavelength of 0.45185 Å. Electrode separation from the cell was performed in an Ar-filled glove box. The amount of sulfur content in all samples was measured by thermogravimetric analysis (TGA, Bruker TG-DTA2000SA) from room temperature to 600 °C at a heating rate of 10 °C min^−1^ in a N_2_ atmosphere.

N_2_ adsorption-desorption isotherms of the synthesized samples were measured at −196 °C with a Micromeritics ASAP 2020 analyser for surface area and porosity. The specific surface areas were determined from nitrogen adsorption using the Brunauer-Emmett-Teller (BET) method in the relative pressure range from 0.05 to 0.3, and total pore volume was determined from the amount adsorbed at the relative pressure of 0.99. Pore-size distribution was evaluated from the adsorption branch of isotherms by the Barrett-Joyner-Halenda (BJH) method. The electrolyte infiltration observations were tested with a contact angle meter using electrolyte (DSA100, Krüss), and the angle was measured by dripping the same amount of electrolyte on the surface of each interlayer. Ultraviolet-visible (UV-Vis) spectroscopy (Agilent Technologies) was used to characterize the adsorption of polysulfide species. Elemental compositions and bonding energies were analysed by X-ray photoelectron spectroscopy (XPS) using an ESCALAB 250 XPS system with a monochromated Al Ka X-ray source. The pore structure for various ILs was measured with a mercury intrusion porosimeter (AutoPore IV 9500 V1.09, Micromeritics Instrument Corporation). The measurements were carried out at 20 °C, while the testing pressure range was between 0.1–60,000 psia.

All the cell fabrication and disassemble were conducted in an Ar-filled glove box (H_2_O  <  0.1 ppm, O_2_  <  0.1 ppm). The assembly order was: cell casing with cathode, gasket 2/3 of the total electrolyte, interlayer, separator, 1/3 of the total electrolyte, Li anode adhered to space, compressing spring, and then cap. The cycled samples for ex situ measurements were obtained by dissembling the cycled Li-S coin cells using a decrimper (Hohsen coin cell disassembling tool) inside Ar-filled glovebox (H_2_O  <  0.1 ppm, O_2_  <  0.1 ppm). The cell components were rinsed using DME repeatedly, then dried in glovebox to remove the solvents. For ex situ XPS measurements, the dried electrodes were first placed into a vacuum transfer vessel in glovebox and then transferred into the XPS ultrahigh vacuum chamber. For ex situ synchrotron X-ray measurements, the samples were sealed with Kapton tape to avoid moisture contamination before measurements. For ex-situ microscopy experiments, Sampling is completed within 1 minute of approaching the instrument and the analysis proceeds.

### Computational details

All structural minimizations were performed with the Vienna ab-initio simulation package^48^. The generalized gradient approximation was used with the Perdew-Burke-Ernzerh of exchange-correlation functionals^49^, and the projector augmented wave^50^ was used to describe core pseudo-potentials. The cutoff energy was set to be 500 eV, and a Monkhorst-Pack 3x3x1 *k*-point mesh was applied. Each calculation was continued until the energy and force convergence criterion of 10^−5 ^eV and 0.02 eV Å^−1^. In graphite systems, the DFT-D3 Grimme method was employed to include the van der Waals interactions^51^. Supercells containing Si (24 atoms) and O (48 atoms) and a vacuum spacing larger than 15 Å were used to model the SiO_2_ (001). The half of the SiO_2_ systems and bottom one carbon layer of 4×4 graphite were fixed during structural optimization. The adsorption energies (E_a_) for Li_2_S_x_ on the surface are defined as E_a_ = E_total_ – E_ps_ – E_surf_, where E_total_ is the energy of the adsorbed system, E_ps_ is the energy of the polysulfides, and E_surf_ is the energy of the bare surface. The nudged elastic band method is used to determine the Li diffusion pathways and migration barriers^52^.

### Reporting summary

Further information on research design is available in the Nature Research Reporting Summary linked to this article.

## Supplementary information

Supplementary Information

Reporting Summary

## Data Availability

The data that support the findings of this study are available from the corresponding authors G.L.X., K.A. and J.-S.Y. upon reasonable request.
