# Supplementary material for: Development of high-energy non-aqueous lithium-sulfur batteries via redox-active interlayer strategy
Source: Nat Commun. 2022 Aug 8;13:4629. doi: 10.1038/s41467-022-31943-8 (PMC9360432; doi:10.1038/s41467-022-31943-8)
Supplement: Supplementary file 1 — Supplementary Information [file 41467_2022_31943_MOESM1_ESM.pdf]

Supplementary Information

Development of high-energy non-aqueous lithium-sulfur batteries via redox-active interlayer strategy

Byong-June Lee[1,9], Chen Zhao[2,9], Jeong-Hoon Yu[1], Tong-Hyun Kang[1], Hyean-Yeol Park[1], Joonhee Kang[3], Yongju Jung[4], Xiang Liu[2], Tianyi Li,[5] Wenqian Xu,[5] Xiao-Bing Zuo[5], Gui-Liang Xu[2*], Khalil Amine[2,6,7*] and Jong-Sung Yu[1,8*]

[1] Department of Energy Science and Engineering, Daegu Gyeongbuk Institute of Science & Technology (DGIST), Daegu, 42988, Republic of Korea

[2] Chemical Sciences and Engineering Division, Argonne National Laboratory, 9700 S Cass Ave, Lemont, IL 60439, United States

[3] Platform Technology Laboratory, Korea Institute of Energy Research, Daejeon 34129, Republic of Korea

[4] Department of Chemical Engineering, Korea University of Technology and Education (KOREATECH), Cheonan, 330-708, Republic of Korea

[5] X-ray Science Division, Argonne National Laboratory, 9700 S Cass Ave, Lemont, IL 60439, United States

[6] Materials Science and Engineering, Stanford University, Stanford, CA, USA

[7] Materials Science, Energy and Nano-engineering Department, Mohammed VI Polytechnic University (UM6P), Ben Guerir, Morocco

[8] Energy Science and Engineering Research Center, DGIST, Daegu, 42988, Republic of Korea

[9] These authors contributed to this equally

Corresponding authors: Dr. Gui-Liang Xu (xug@anl.gov); Dr. Khalil Amine (amine@anl.gov) and Prof. Jong-Sung Yu (jsyu@dgist.ac.kr)

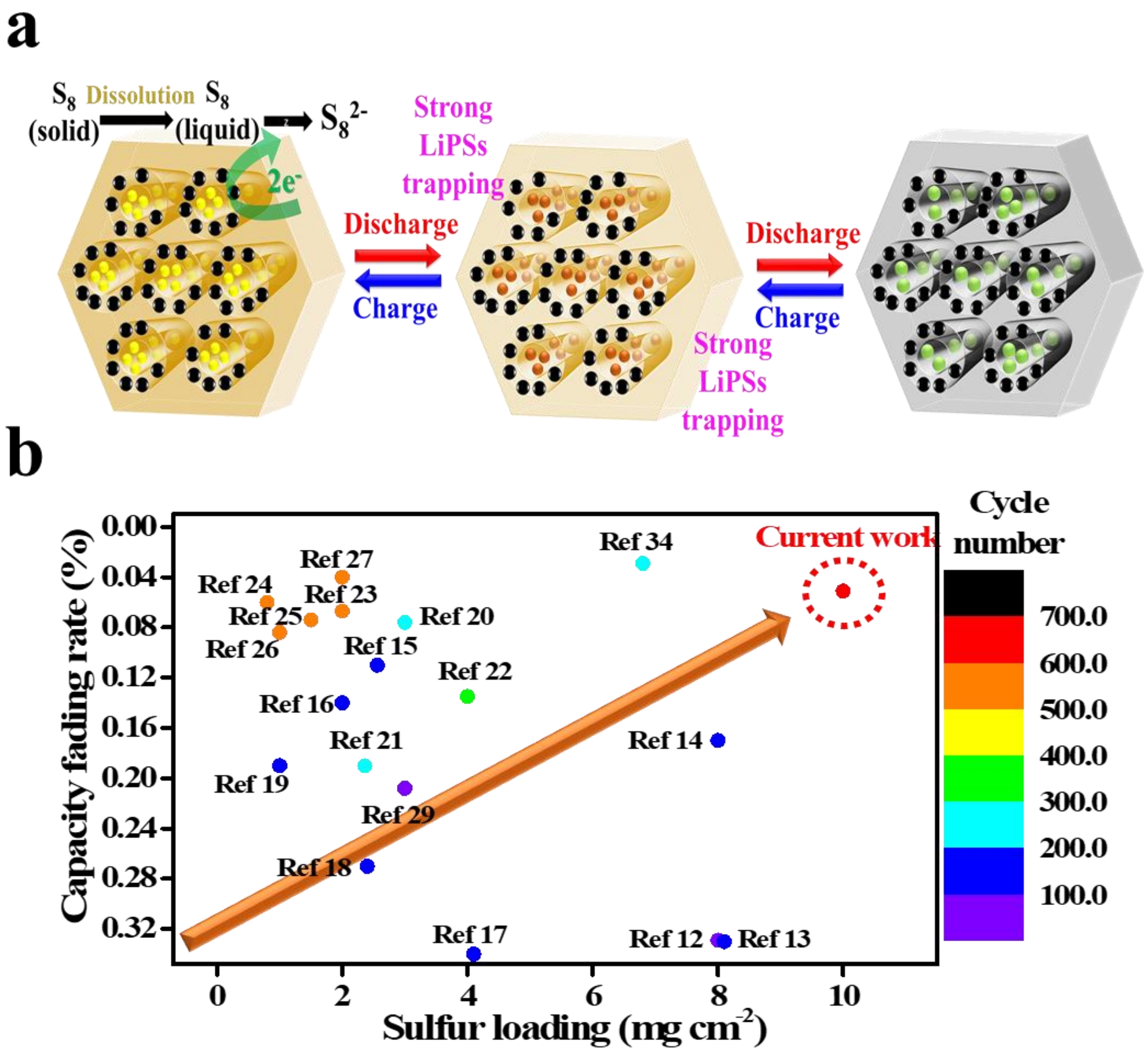

Supplementary Figure 1 (a) Operation principle of Li-S battery with S-containing polar IL. (b) Summary of areal sulfur loading and cycle stability for current work and recent reports in the literature. Ref number indicates reference cited in the Supplementary References List.

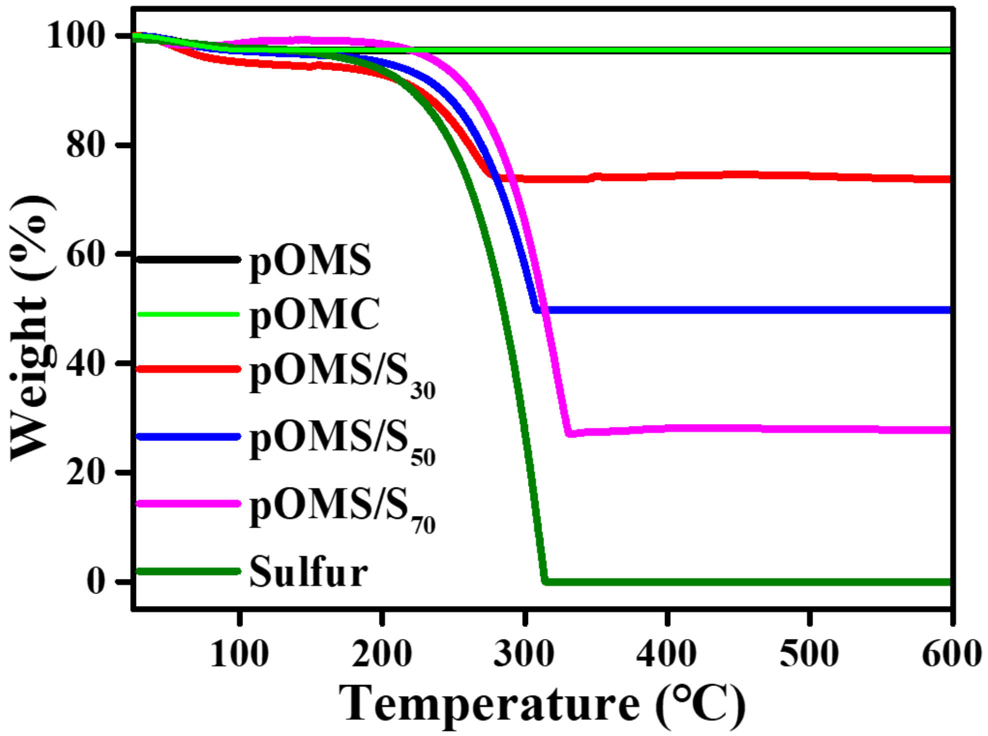

Supplementary Figure 2 | TGA curves of the bare sulfur powder and pOMC, pOMS, and pOMS/S_x composites under the nitrogen atmosphere.

The TGA curve for bare sulfur powder displays one weight loss stage from 200 to 350 °C. Interestingly, the TGA decay curves shift to slightly higher temperature in the pOMS/S_x composites relative to the bare sulfur, probably indicating the strong confinement effect of sulfur in the porous pOMS framework. The flat line over 350 °C shows the complete evaporation of sulfur from the pOMS/S_x composites, from which the sulfur weight percentage was determined to be 30.5, 50.4, and 70.2 wt. % for pOMS/S_{30}, pOMS/S_{50}, and pOMS/S_{70} composites, respectively.

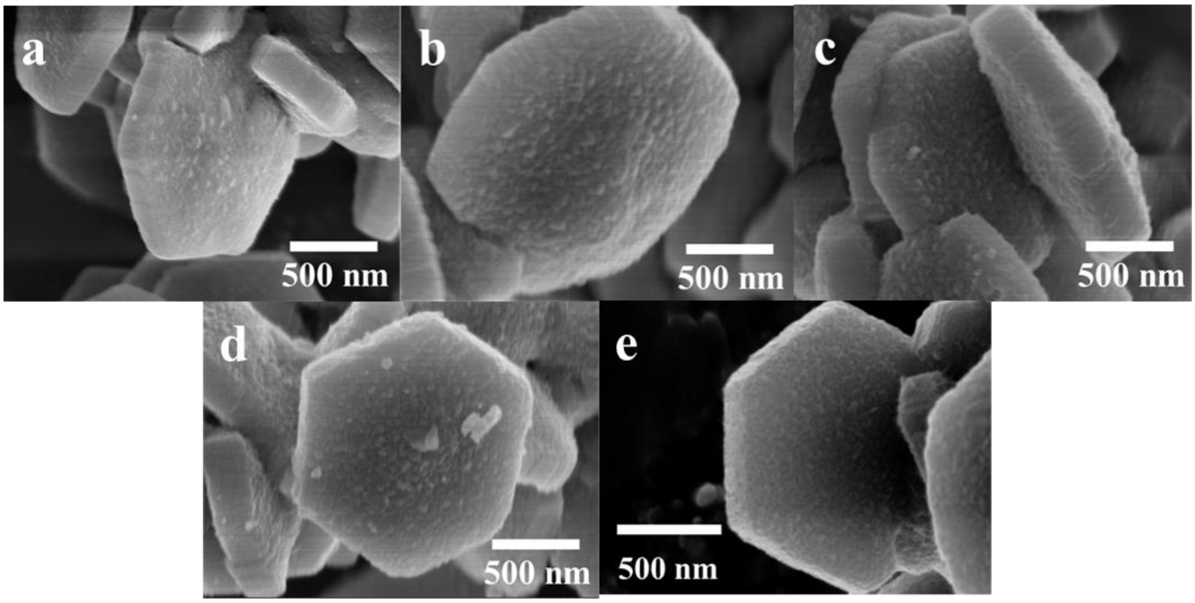

Supplementary Figure 3 | SEM images of (a) pOMS, (b) pOMS/S_{30}, (c) pOMS/S_{50}, (d) pOMS/S_{70}, and (e) pOMC.

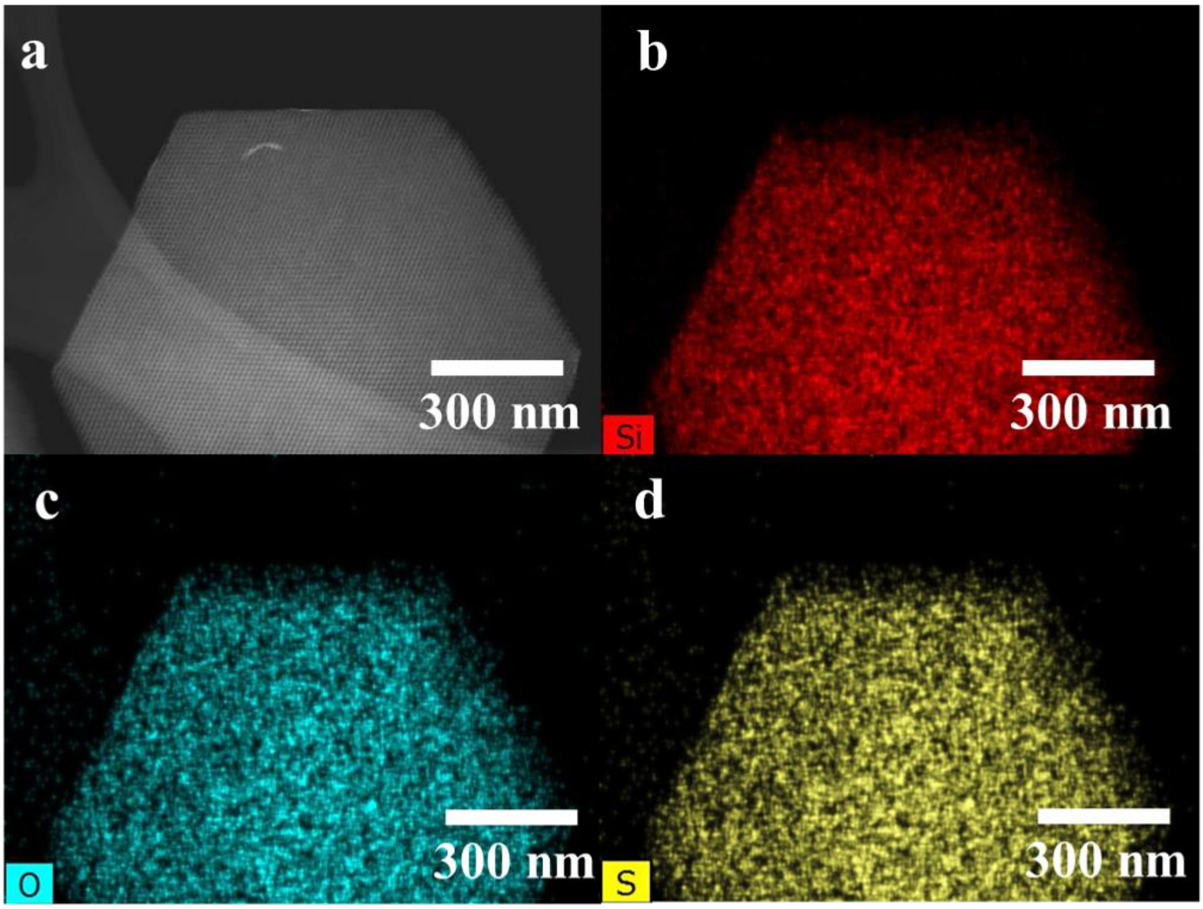

Supplementary Figure 4 | HAADF-STEM image of (a) pOMS/S_{50}, and the corresponding elemental mappings of (b) silicon, (c) oxygen, and (d) sulfur.

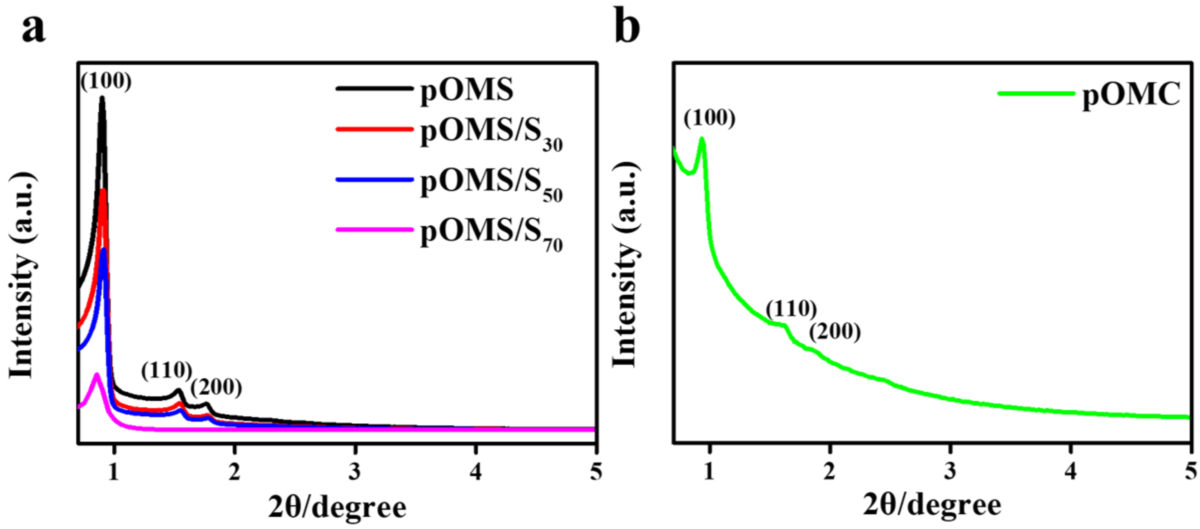

Supplementary Figure 5 | SAXRD patterns of (a) pOMS/S_x composites along with bare pOMS and (b) pOMC.

The pristine pOMS and pOMC exhibit diffraction peaks at around 2θ = 0.90, 1.52, and 1.76° corresponding to an intense (100) and two weaker (110) and (200) diffraction peaks, respectively, which are characteristic of a 2D hexagonal p6mm pore structure[1]. With increasing sulfur loading in pOMS, the intensities of the (100), (110), and (200) diffraction peaks gradually decrease for pOMS/S_{30} and pOMS/S_{50} composites. Finally, for pOMS/S_{70}, the (100) peak alone remains small, and the (110) and (200) peaks disappear, indicating that mesopores are gradually filled with more sulfur in sequence.

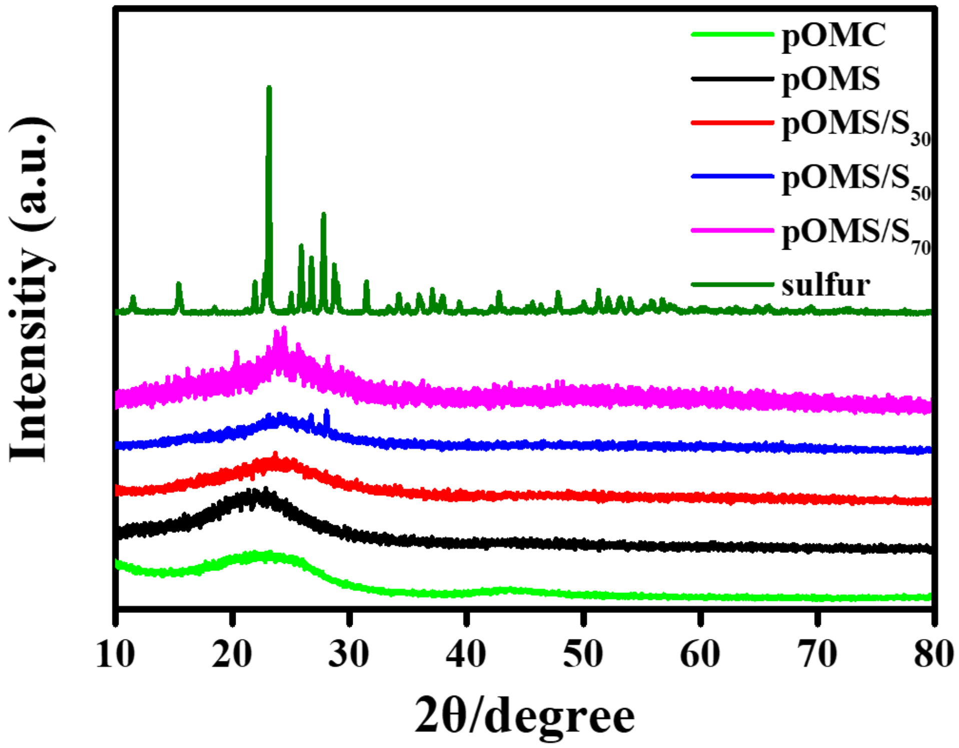

Supplementary Figure 6 | WAXRD patterns of pOMS/S_x composites along with bare pOMS and pOMC.

The WAXRD pattern of sulfur indicates an orthorhombic structure as a crystalline phase of S (JCPDS card No. 01-078-1889). WAXRD shows only a broad amorphous peak at $2\theta = 22°$ for bare pOMS and pOMC[1]. For the pOMS/S_{30} composite, the WAXRD pattern exhibits only a broad signal at around $2\theta = 24°$, which is typical of amorphous silica without the diffraction peaks for sulfur, indicating that sulfur is mostly impregnated into the pores of pOMS. However, for pOMS/S_{50} and pOMS/S_{70}, the sulfur diffraction peaks are weakly observed due to the presence of some crystallized sulfur on the surface of pOMS with increasing sulfur content.

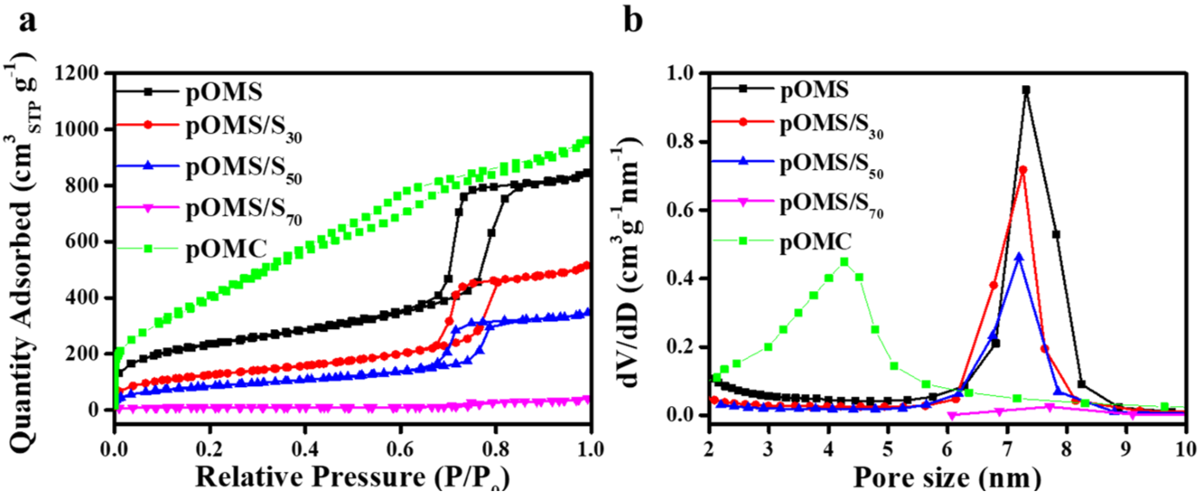

Supplementary Figure 7 | (a) Nitrogen adsorption/desorption isotherms and (b) pore size distribution curves of the pOMS, pOMC, and various pOMS/S_x composites.

The pristine pOMS, pOMS/S_{30}, and pOMS/S_{50} composites exhibit type IV isotherms with a large hysteresis loop, which is typical of a uniform mesoporous structure[1] (Supplementary Figure 7a). On the other hand, the pOMS/S_{70} composite has a sharp reduction of a hysteresis loop due to the near complete filling of sulfur in the mesochannels of the pOMS (Supplementary Figure 7a). The pOMS reveals a high BET surface area of 834 m^2 g^{-1} and a large pore volume of 1.36 cm^3 g^{-1} along with a narrow pore size distribution centered at 7.4 nm (Supplementary Figure 7b). With an increase in sulfur infiltration, the BET surface area decreases drastically to 451, 303, and 33 m^2 g^{-1} along with total pore volumes of 0.88, 0.58, and 0.08 cm^3 g^{-1} for pOMS/S_{30}, pOMS/S_{50}, and pOMS/S_{70}, respectively. Interestingly, the pore size distribution remains nearly identical even after sulfur infiltration, while its intensity is drastically decreased (Supplementary Figure 7b). Such a decrease in surface properties indicates that sulfur successfully infiltrates the pores in the pOMS matrix. The pOMC also displays type IV isotherms and shows a much higher specific surface area[2] of 1,544 m^2 g^{-1} and a small pore size distribution of 4.3 nm along with a high total pore volume of 2.98 cm^3 g^{-1}.

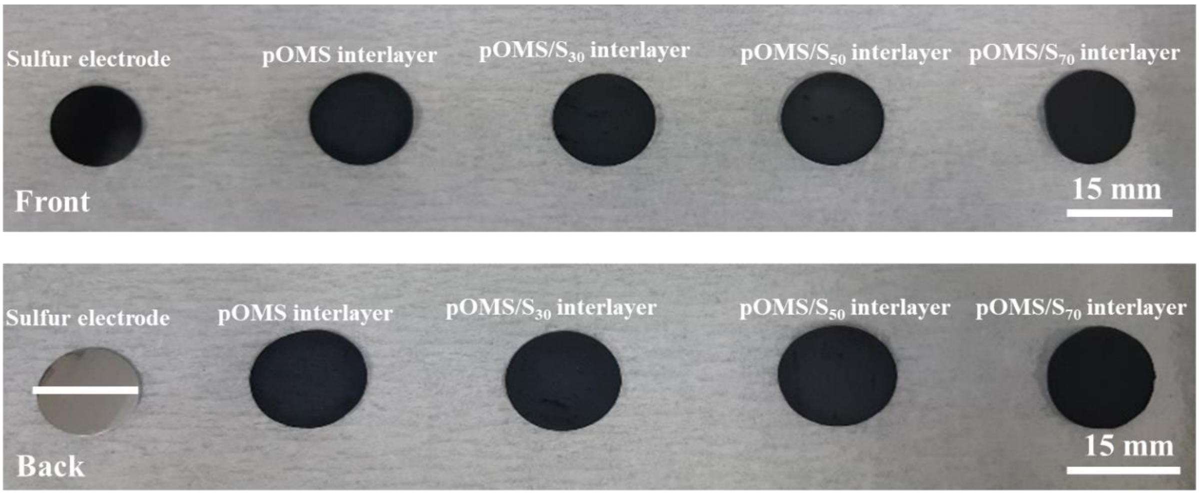

Supplementary Figure 8 | Photographic pictures of sulfur electrode, pOMS IL, and different pOMS/S_x ILs.

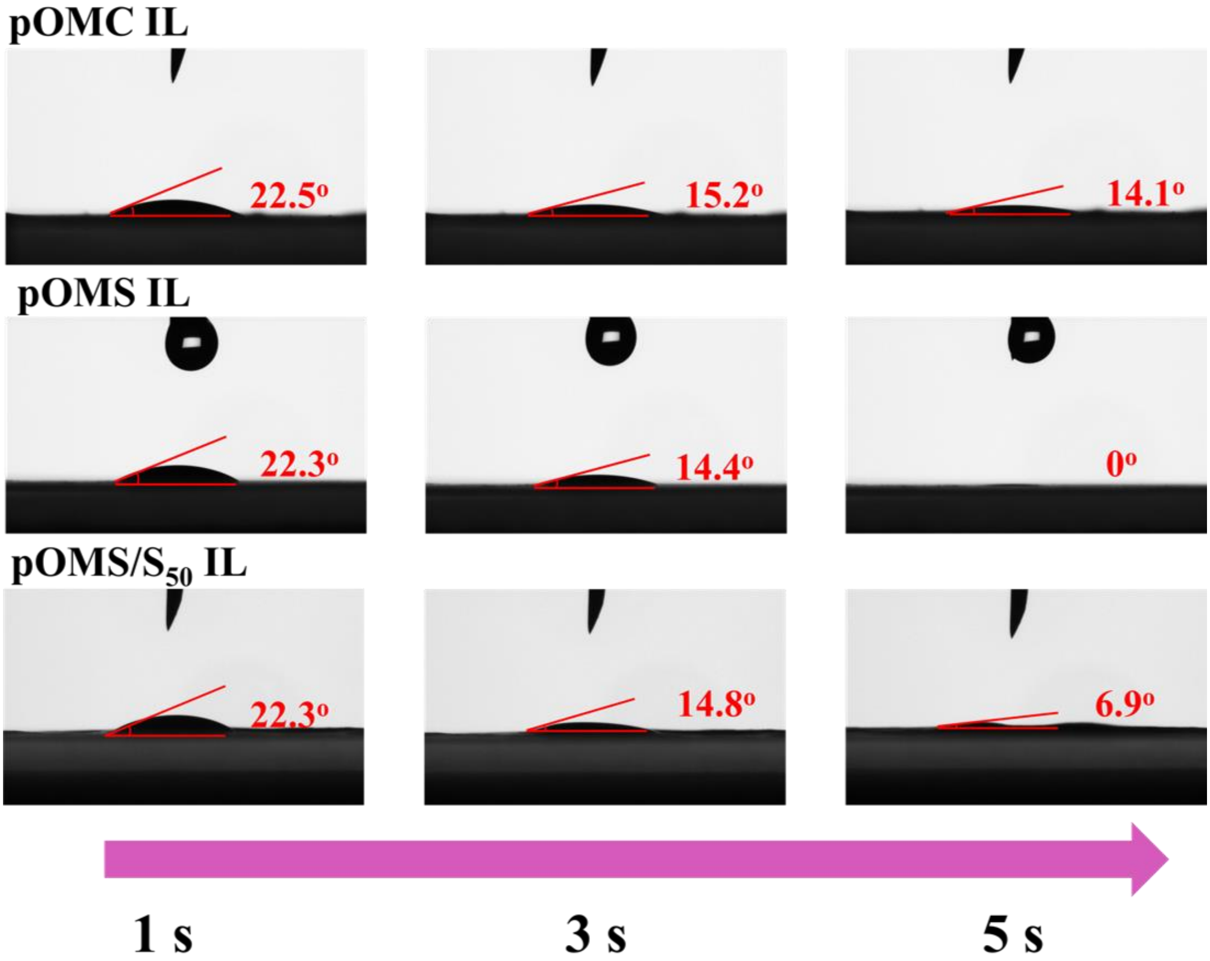

Supplementary Figure 9 | Contact angle measurements of the electrolyte (1.0 M LiTFSI dissolved in DME/DOL in a 1:1 volumetric ratio with 0.2 M $LiNO_3$ as an additive) on the surface for pOMC, pOMS, and pOMS/S_{50} ILs with electrolyte after 1, 3, and 5 s.

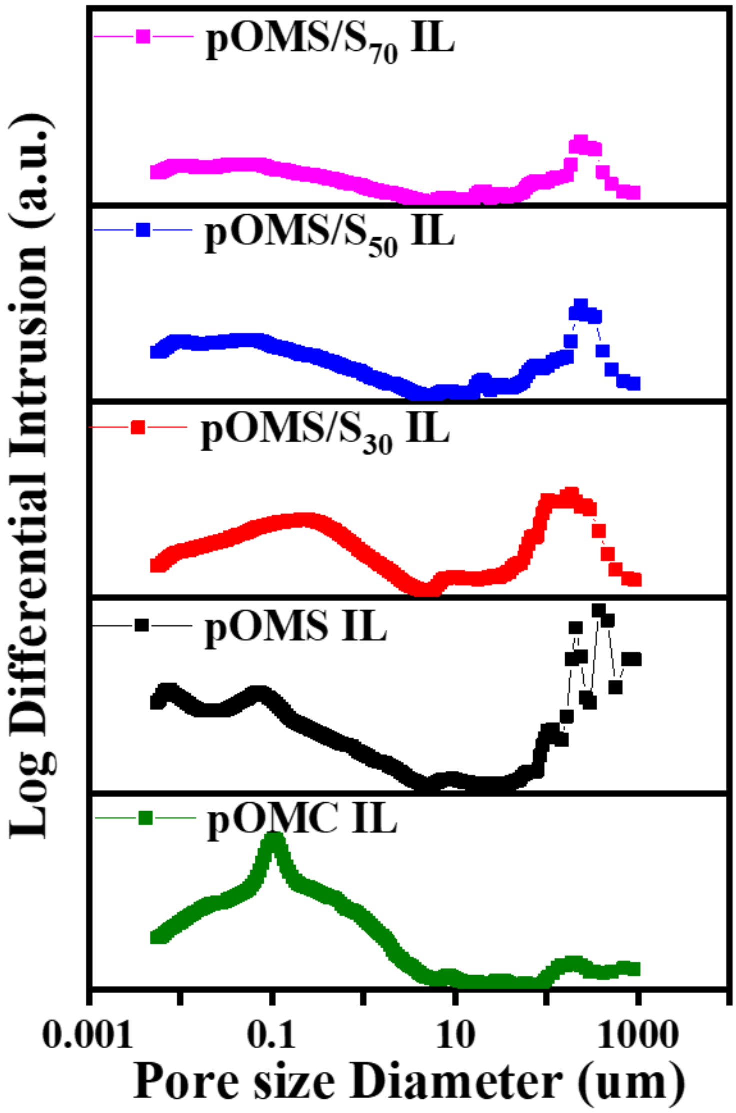

Supplementary Figure 10 | The pore size distribution curves of pOMC, pOMS, and different pOMS/S ILs determined by mercury intrusion.

The mercury intrusion characterization has been adopted to investigate the pore structure of various ILs. Since pOMC has excellent porous structure, the pOMC IL has a high porosity of 76 % and total intrusion volume of 1.52 cm^3 g^{-1}, which is larger than the porosity and total intrusion volume of pOMS IL (74 % and 1.31 cm^3 g^{-1}). However, with embedded sulfur in IL, the porosity and total intrusion volume decrease in the pOMS IL samples.

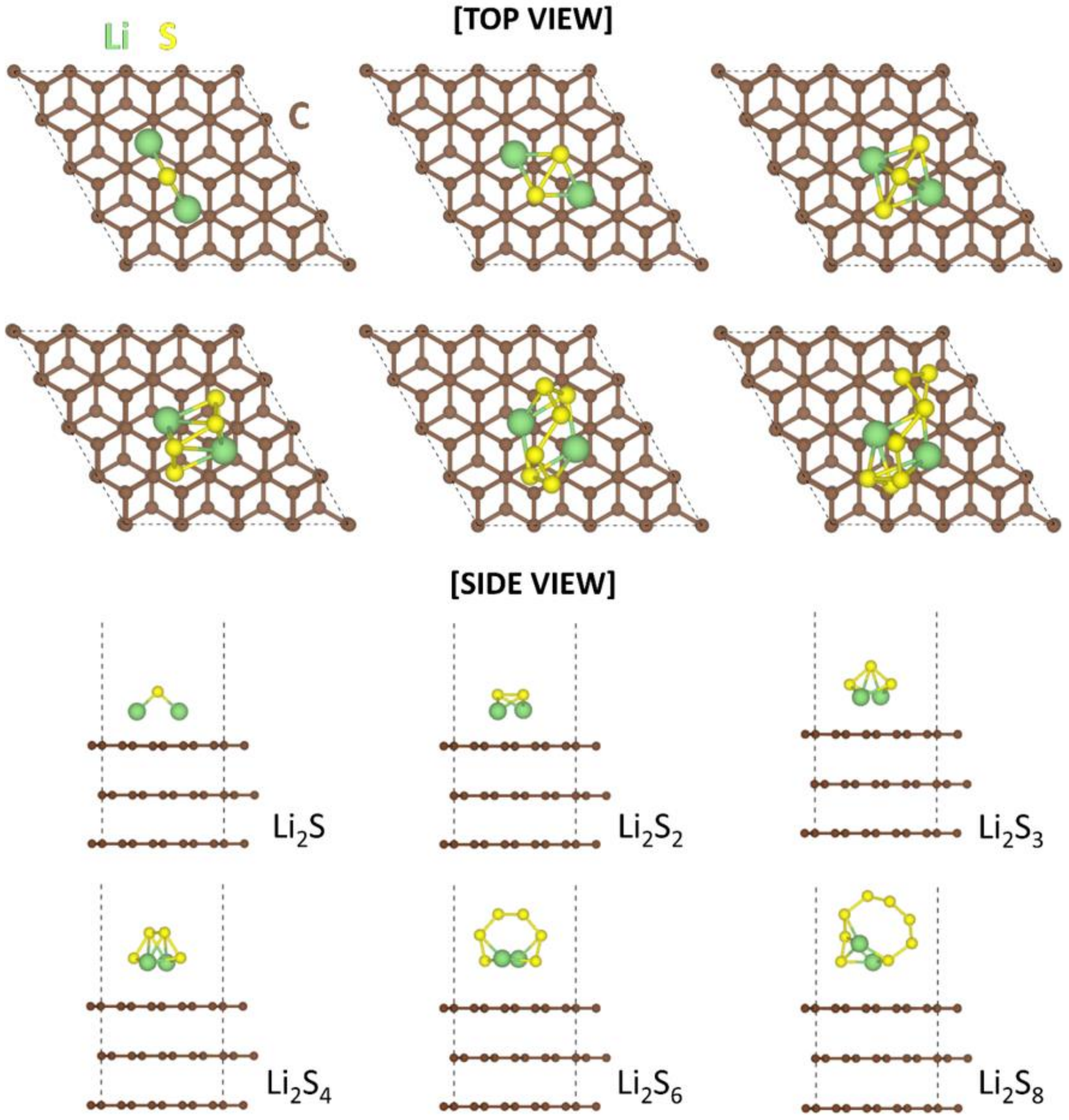

Supplementary Figure 11 | Optimized structures for Li_2S_x adsorption on graphite (001) surface. The green, yellow, and brown balls represent lithium, sulfur, and carbon atoms, respectively.

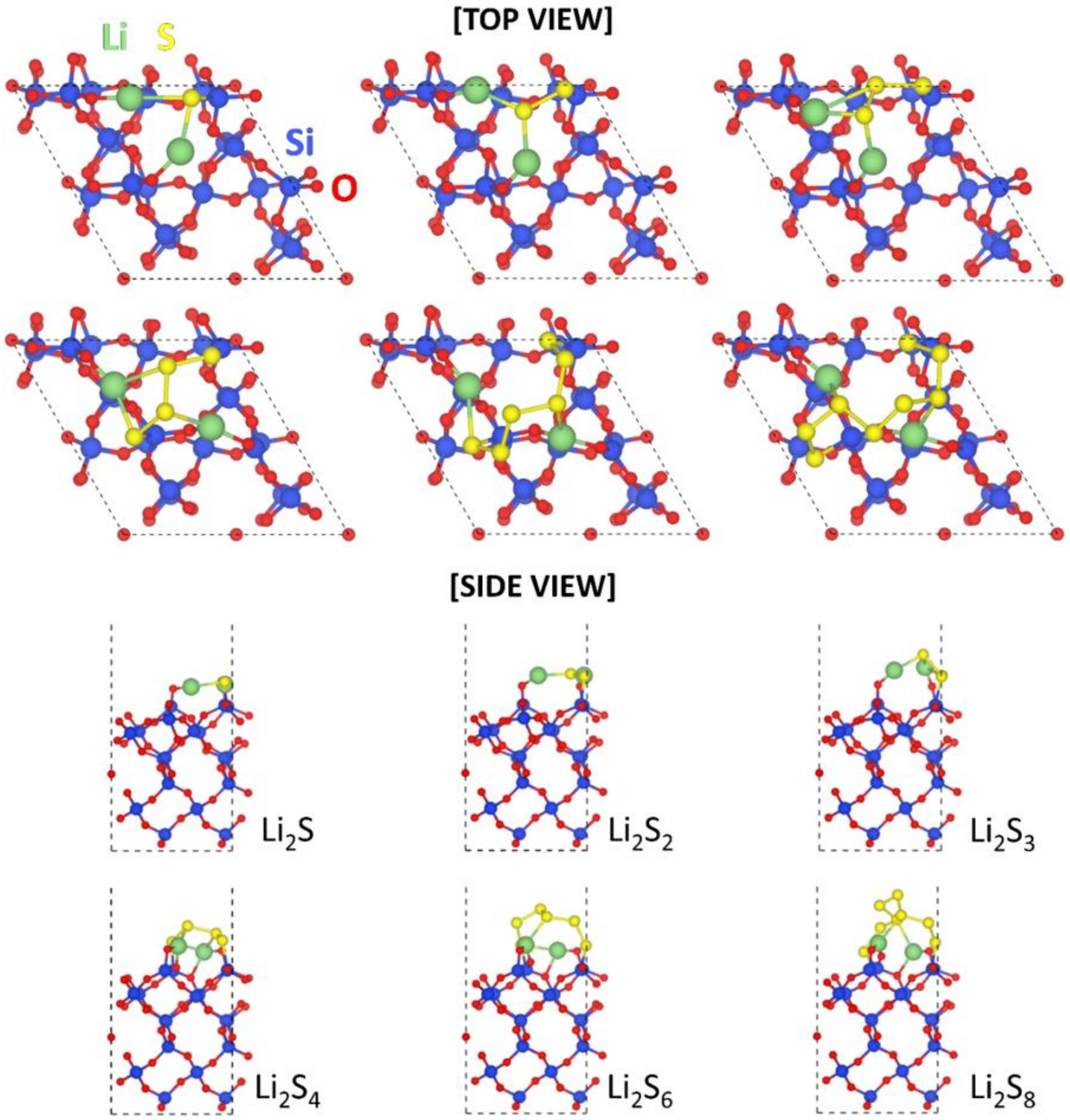

Supplementary Figure 12 | Optimized structures for Li_2S_x adsorption on SiO_2 (001) surface. The green, yellow, blue, and red balls represent lithium, sulfur, silicon, and oxygen atoms, respectively.

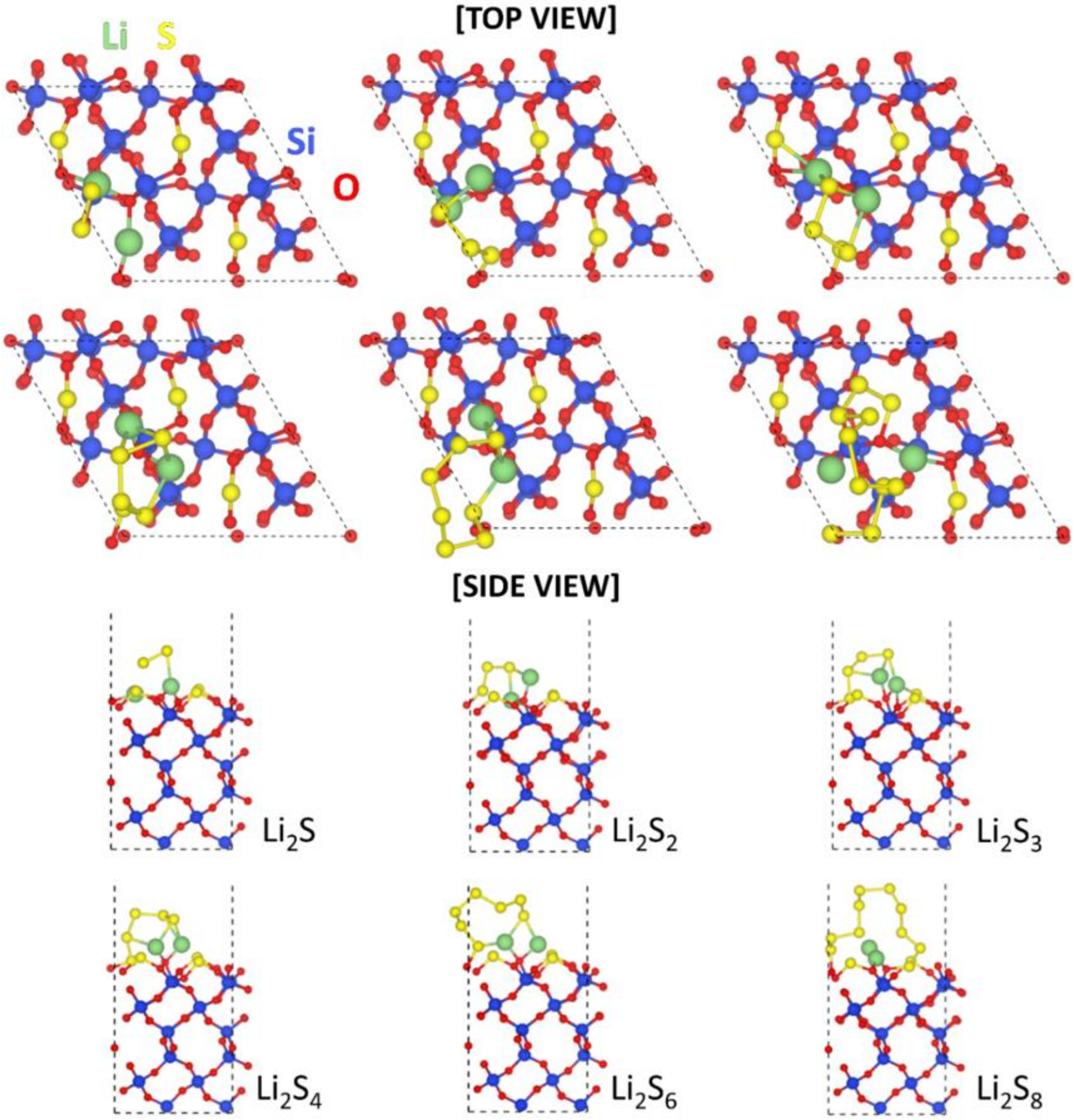

Supplementary Figure 13 | Optimized structures for Li_2S_x adsorption on S-containing SiO_2 (001) surface. The green, yellow, blue, and red balls represent lithium, sulfur, silicon, and oxygen atoms, respectively.

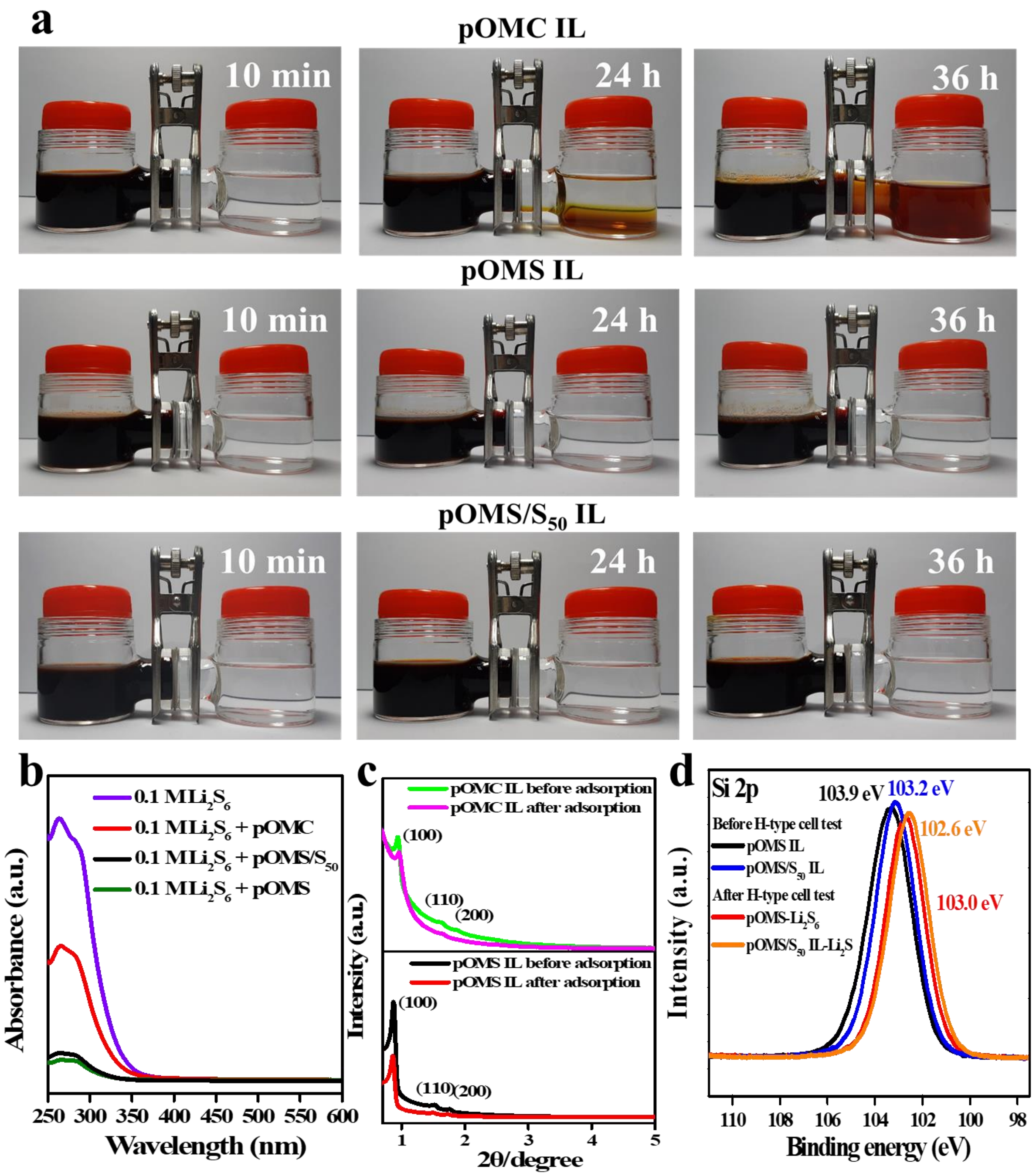

Supplementary Figure 14 | a, Digital photographs of a H-type cell with 0.1 M Li_2S_6 in DME/DOL solution (left chamber) and pure DME/DOL solvent (right chamber) separated by pOMC IL (top panel), pOMS IL (middle panel), or pOMS/S_{50} IL (bottom panel). b, UV-vis absorption spectra of the right chamber solution for pOMC, pOMS, and pOMS/S_{50} ILs after visual LiPSs diffusion test. c, SAXRD patterns of pOMC and pOMS ILs before and after H-type cell test. d, High-resolution Si 2p XPS spectra of pOMS and pOMS/S_{50} ILs before and after H-type cell test.

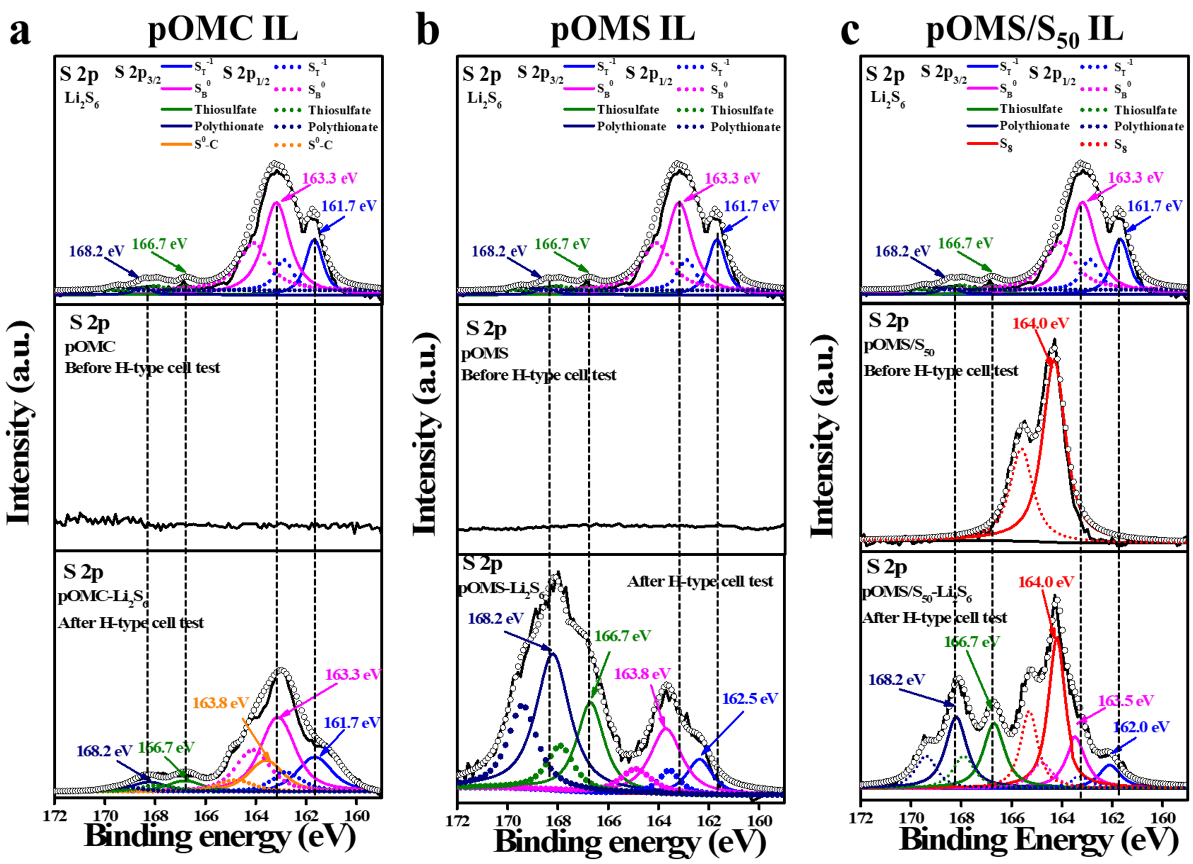

Supplementary Figure 15 | High-resolution S 2p XPS spectra before and after H-type cell test for (a) pOMC, (b) pOMS, and (c) pOMS/S$_{50}$, respectively. (Black line: original data, open circle: overall fitted data, solid S 2p$_{3/2}$ and dotted S 2p$_{1/2}$ lines: fitted individual spectra.)

To examine the interaction of pOMC, pOMS, or pOMS/S$_{50}$ IL with LiPSs, XPS was employed after the H-type cell tests. For the Li_2S_6, the S 2p3/2 spectrum shows two pairs of peaks located at 161.7 and 163.3 eV ascribed to the terminal sulfur (S_T^{-1}, Li-S) and bridging sulfur (S_B^0, S-S)[1], respectively. In addition, two weak peaks at 166.7 and 168.2 eV for Li_2S_6 correspond to the oxidized sulfur species, which are assigned to the thiosulfate (O_3S-S) and polythionate complex (O_3S-S_x-S), respectively, probably due to the brief exposure to oxygen in the air during sample transfer. After Li_2S_6 adsorption in the pOMC, both S_T^{-1} and S_B^0 peaks show little change in the binding energy. However, a new weak peak appears at 163.8 eV, corresponding to the C-S species[3] (Supplementary Figure 15a). On the other hand, both the S_T^{-1} and S_B^0 peaks shift to higher binding energy positions at 162.5 eV (S_T-pOMS) and 163.8 eV (S_B-pOMS) for pOMS, respectively, compared to S_T^{-1} and S_B^0 in S 2p$_{3/2}$ of Li_2S_6. Furthermore, the thiosulfate and polythionate signals grow as a strong broad peak in the range of 165-172 eV, which can originate from a strong interaction between the oxygen-rich surface of the pOMS and sulfur of the Li_2S_6 (Supplementary Figure 15b). For pOMS/S$_{50}$

without adsorption of Li_2S_6, two major peaks are located at 164 and 165.2 eV, corresponding to S $2p_{3/2}$ and S $2p_{1/2}$[4]. After adsorption of Li_2S_6 in the pOMS/S_{50}, the S 2p spectrum exhibits two higher binding energies at 162 (S_T-pOMS/S_{50}) and 163.50 eV (S_B-pOMS/S_{50}), compared to the pure Li_2S_6. In addition, the thiosulfate and polythionate signals also increase significantly as in the result for pOMS-Li_2S_6 (Supplementary Figure 15c). The results imply that polar pOMS and pOMS/S_{50} ILs can provide efficient trapping sites for LiPSs during the reaction in Li-S batteries.

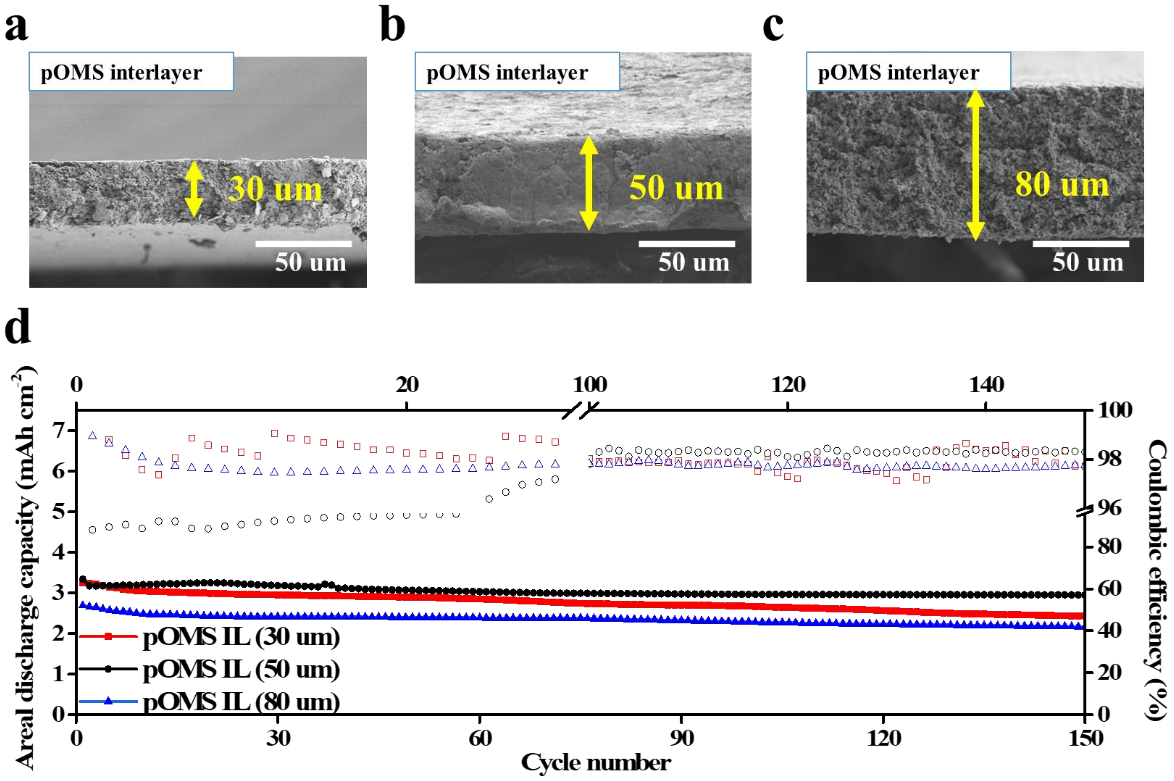

Supplementary Figure 16 | Cross-sectional SEM images of pOMS IL with different thicknesses of (a) 30, (b) 50, and (c) 80 μm, respectively. Cycling performances of the cells with different thicknesses of pOMS ILs at a charge/discharge rate of 335 mA g^{-1}.

To evaluate the optimized thickness of the IL, the pOMS ILs were prepared in varying thicknesses of 30, 50, and 80 μm. Due to the relatively rapid electrolyte permeation into the IL, the 30 μm pOMS IL showed the high initial areal discharged capacity, but illustrated the faster capacity decay owing to weak adsorption capability of relatively thin pOMS IL for LiPSs. The 80 μm pOMS IL exhibited excellent cycle stability due to the higher adsorption capability of thick IL and, but illustrates the low areal capacity due to the relatively slow electrolyte permeation into the IL. Among the three ILs, the pOMS IL with 50 μm thickness exhibits excellent cycle performance due to its optimum adsorption capability for polysulfide and electrolyte permeation low Li ion diffusion. Therefore, this current work focused on the 50 μm pOMS IL.

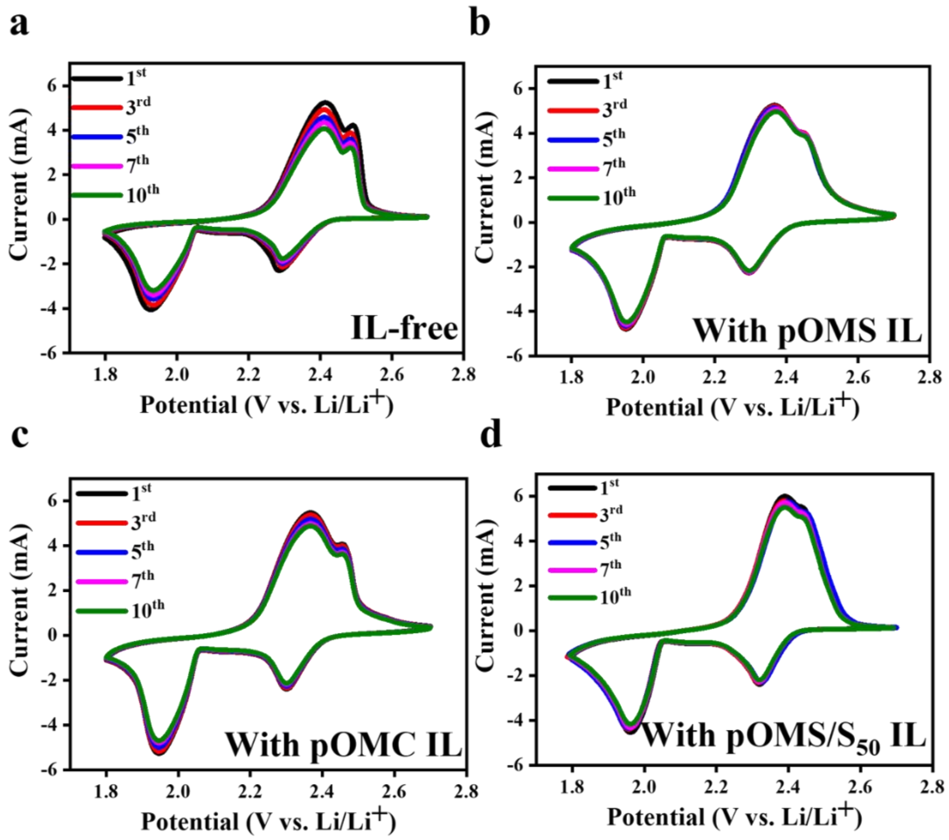

Supplementary Figure 17 | CV curves of (a) IL-free, (b) pOMS IL, (c) pOMC IL, and (d) pOMS/S_{50} IL cells for the first 10 cycles at a scan rate of 0.2 mV s^{-1} from 1.8 to 2.7 V (vs. Li/Li^+).

For a cell with pOMS IL, two cathodic peaks at 2.30 and 1.95 V were observed, corresponding to the transformation of solid sulfur to highly soluble long-chain LiPSs (Li_2S_n, $4 < n \leq 8$) and the subsequent reduction towards insoluble solid Li_2S_2 and Li_2S[5], respectively (Supplementary Figure 17b). The two anodic peaks at 2.35 and 2.43 V are related to the oxidation reaction from Li_2S/Li_2S_2 to soluble LiPSs and eventually to solid sulfur, respectively. Compared with the CV curves for the IL-free and pOMC IL cells (Supplementary Figures 17a and c), the pOMS IL cell exhibits no change in the CV peaks for the first 10 cycles, indicating excellent cycle stability and highly reversible redox reactions in Li-S battery with the pOMS IL. In addition, the pOMS/S_{50} IL cell (Supplementary Figure 17d) shows the peak shift in CV compared to the pOMS IL cell due to high sulfur loading. However, it shows excellent reversibility despite high sulfur loading. The result strongly suggests that pOMS/S_{50} can be successfully used as an IL to confine the sulfur and LiPSs, giving excellent cycle stability.

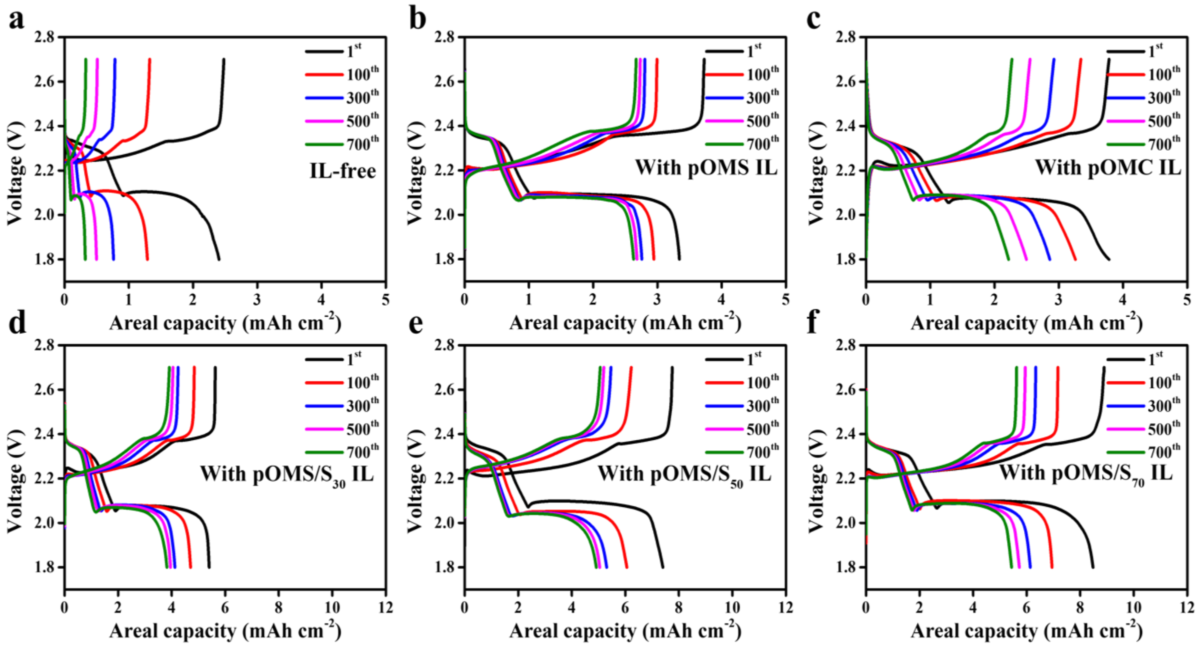

Supplementary Figure 18 | Galvanostatic charge-discharge profiles of the (a) IL-free (b) pOMS IL, (c) pOMC IL, (d) pOMS/S_{30} IL, (e) pOMS/S_{50} IL, and (f) pOMS/S_{70} IL cells at 335 mA g^{-1} and 25 ^{o}C for 700 cycles under E/S ratio of 10 µl mg^{-1}.

The first plateau is corresponding to the transformation of S_8 to long-chain polysulfides, while the second plateau is related to further reduction of long-chain polysulfides to Li_2S. In the IL-free cell, the long-chain polysulfides are very easily dissolved in the electrolytes, leading to continuous loss of active materials. Therefore, the second plateau, i.e., the reduction of long-chain polysulfides to Li_2S is shortened due to decrease of the active polysulfide concentration, leading to low sulfur utilization.

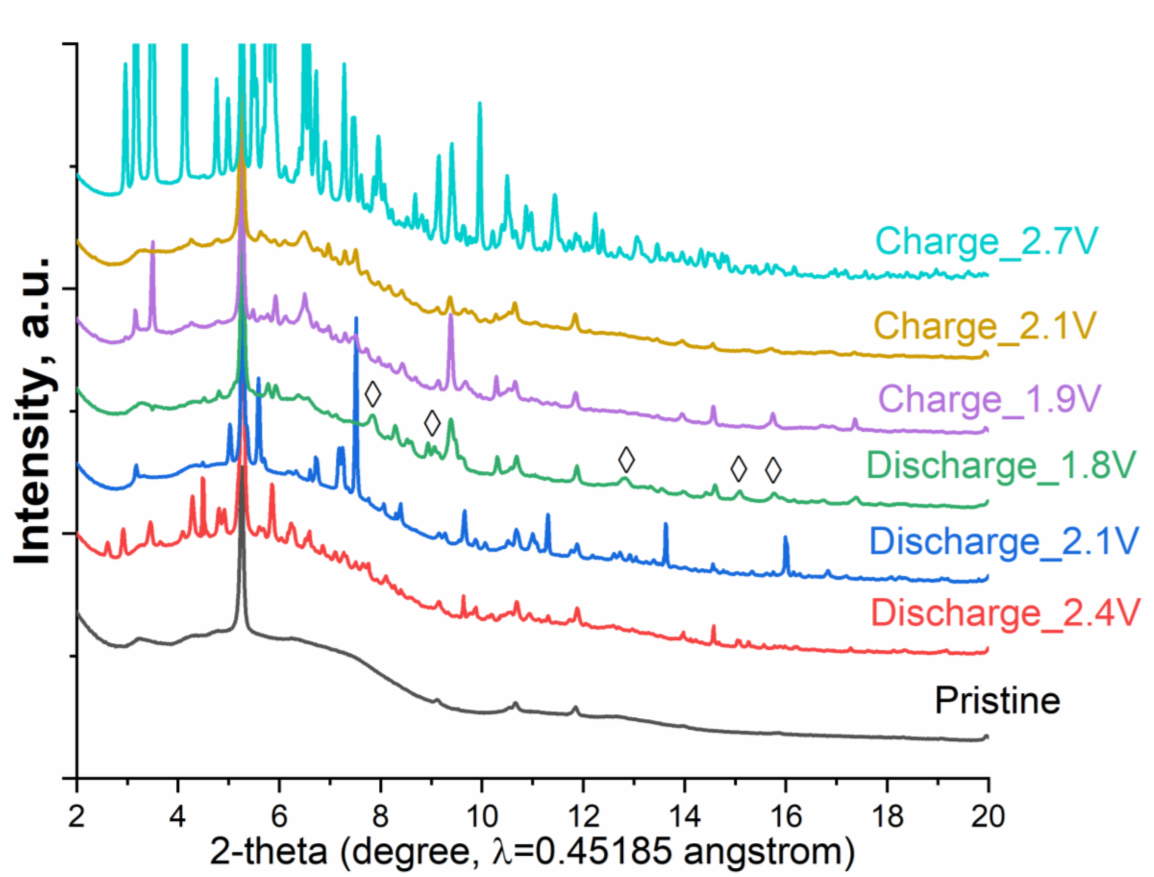

Supplementary Figure 19 | HEXRD patterns of pOMS/S_{50} IL at different charge/discharge states. The diamond symbols in the XRD pattern of discharge_1.8V represent Li_2S.

As shown, after discharge, numerous diffraction peaks appeared, which should be related to the trap of dissolved polysulfides from the bare sulfur cathode. When further discharge to 1.8V, the diffraction peaks of Li_2S can be observed, confirming that the S-containing interlayer not only trap polysulfides, but also contribute to the redox reaction of embedded sulfur and trapped polysulfides. During the charge process, we observed a significant increase of diffraction peaks when charged to 2.7 V, which should be due to the severe polysulfides shuttle during charge of bare sulfur cathode, while the pOMS/S_{50} IL could well confine these polysulfides due to their strong binding strength towards polysulfides.

In general, the HEXRD results confirmed that S-containing interlayer could trap polysulfides and involve redox reaction of sulfur during charge/discharge.

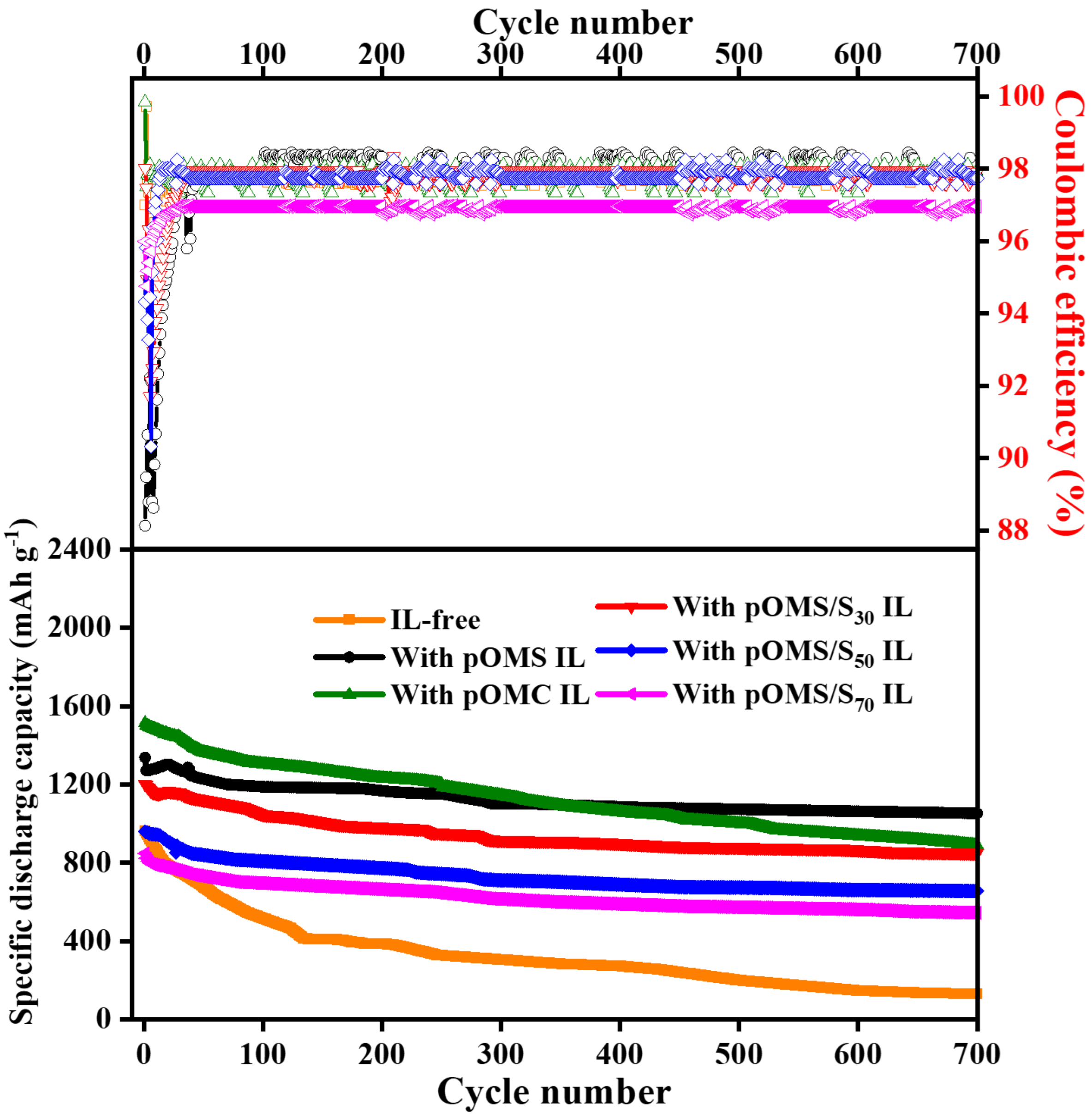

Supplementary Figure 20 | Specific discharge capacity curves measured for cells with IL-free and different ILs at 335 mA g^{-1} and 25 ^{o}C for 700 cycles under an E/S ratio of 10 µl mg^{-1}.

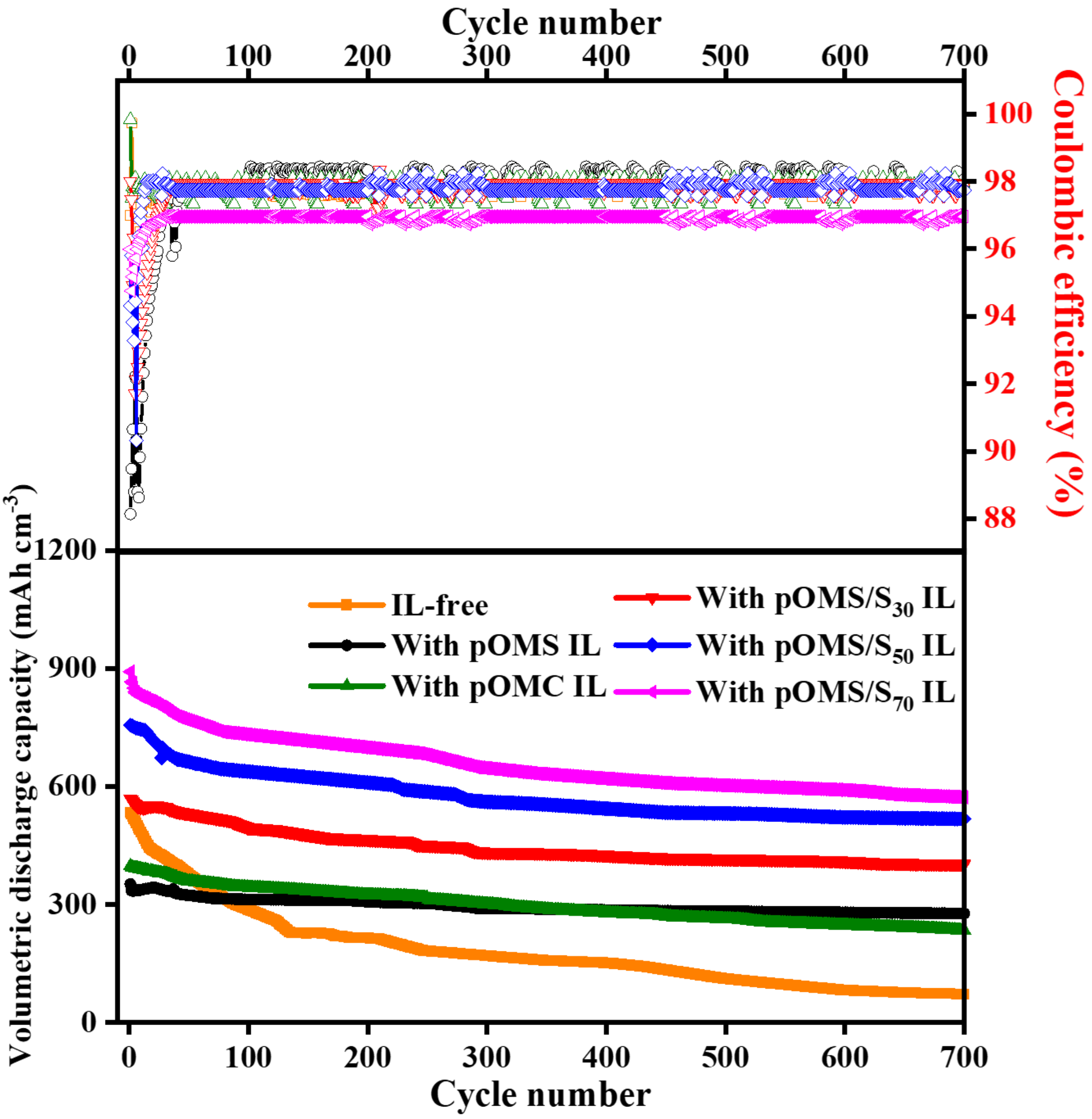

Supplementary Figure 21 | Volumetric capacity curves measured for the cells with IL-free and different ILs at 335 mA g^{-1} and 25 ^{o}C for 700 cycles under an E/S ratio of 10 µl mg^{-1}.

Calculation for areal and volumetric capacities: The areal capacity was obtained from equation (2) while the volumetric capacity was calculated from equation (3).

Areal capacity (mAh cm^{-2}) = Specific capacity (mAh g^{-1}) × sum of cathode and IL sulfur loading (g cm^{-2}) (2)

Volumetric capacity (mAh cm^{-3}) = areal capacity (mAh cm^{-2}) / sum of cathode and IL thicknesses (cm) (3)

The cathode and interlayer have the thicknesses of 45 and 50 um, respectively. Since pOMS and pOMC IL do not contain sulfur in the thick IL, the pOMS IL and pOMC IL cells show lower volumetric capacity than that of IL-fee cell. However, the cells with sulfur-containing pOMS/S ILs have a higher areal capacity, and thus a higher volumetric capacity than IL-fee cell. The result suggests that pOMS/S IL with high sulfur loading can enhance volumetric capacity and improve cycle stability.

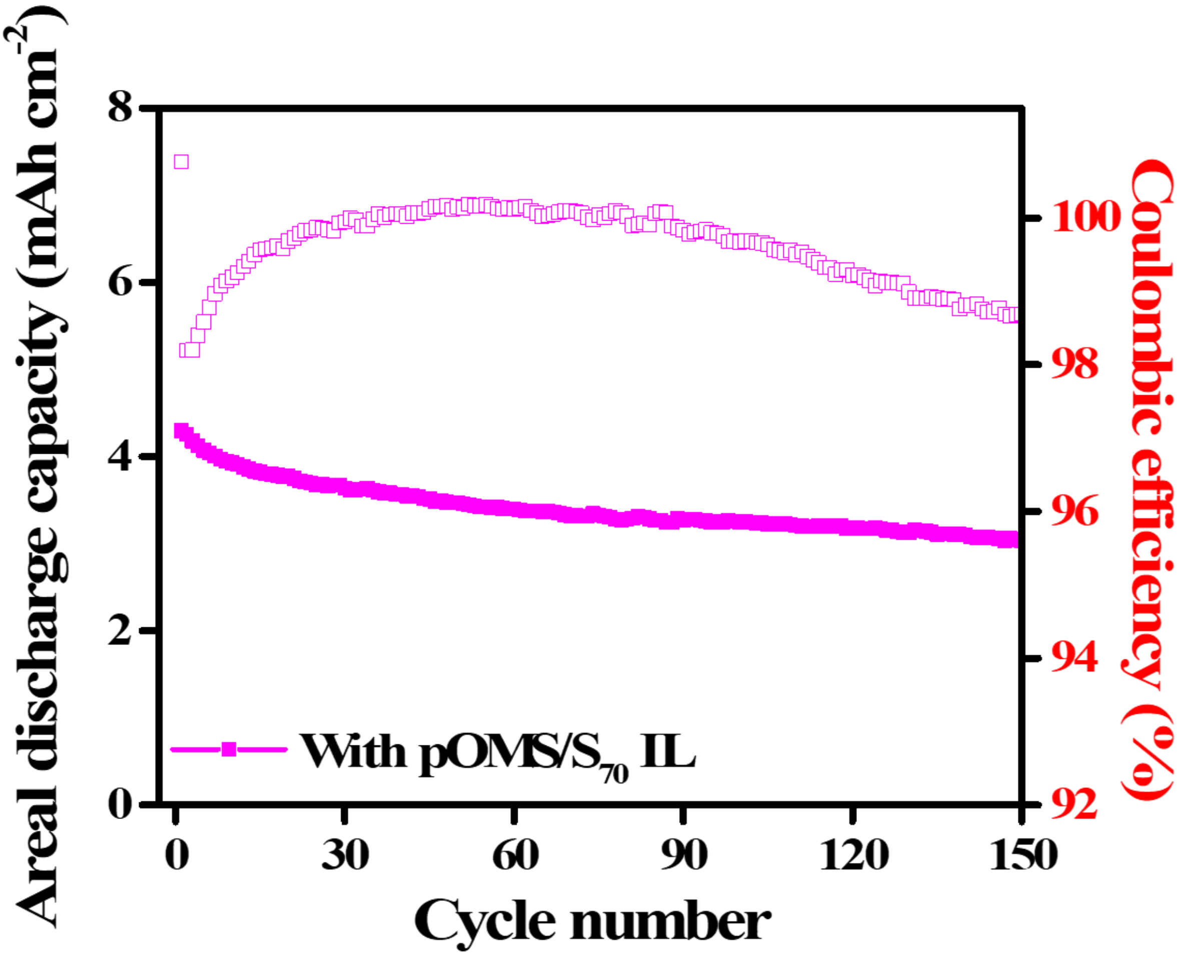

Supplementary Figure 22 | High-rate cycle performances of a cell prepared with pure sulfur cathode with pOMS/S_{70} IL cell at a charge/discharge specific current of 3,350 mA g^{-1} and 25 °C for 150 cycles under an E/S ratio of 10 µl mg^{-1}.

To confirm the electrochemical performance for pOMS/S_{70} IL at high specific current, the cycling performance of pOMS/S_{70} IL cell was measured for 150 cycles at a higher charge/discharge specific current of 3,350 mA g^{-1}. The pOMS/S_{70} IL cell shows initial areal capacity of 4.3 mAh cm^{-2}. After 150 cycles, the reversible areal discharge capacity retains 3.0 mAh cm^{-2}, corresponding to capacity retention of 71 %. The result indicates that sulfur-containing pOMS IL can mitigate the polysulfides migration despite the high sulfur loading in the IL and high charge/discharge rate.

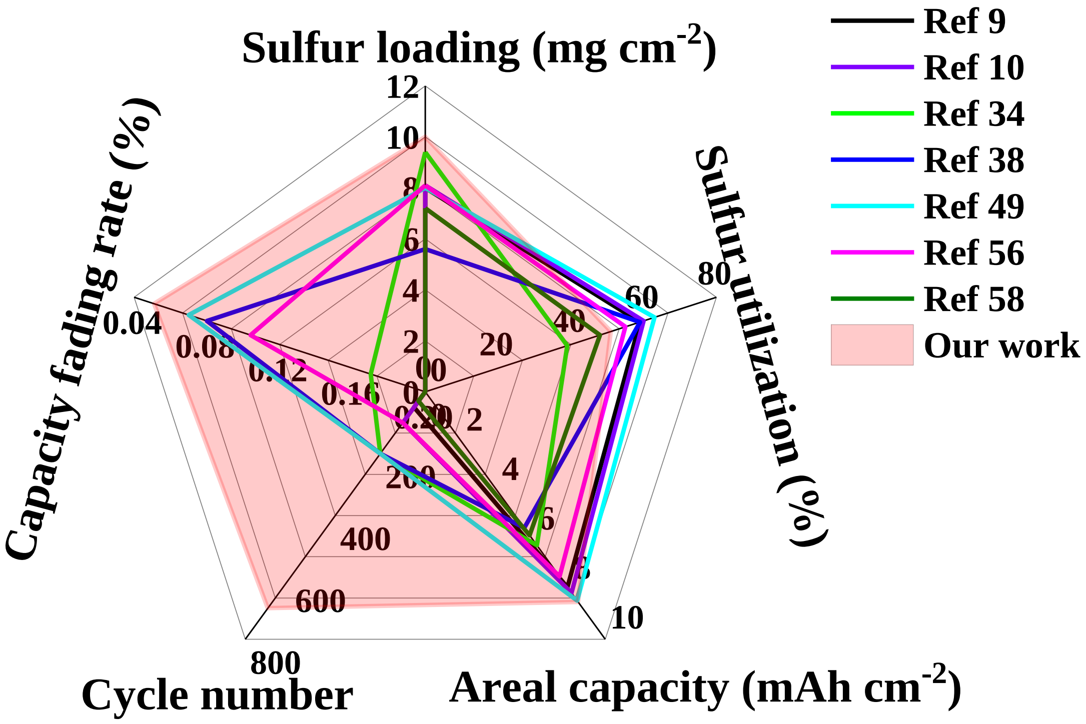

Supplementary Figure 23 | Performance comparison between the current cell (pure sulfur cathode with pOMS/S_{70} IL) and previously reported Li-S cells.

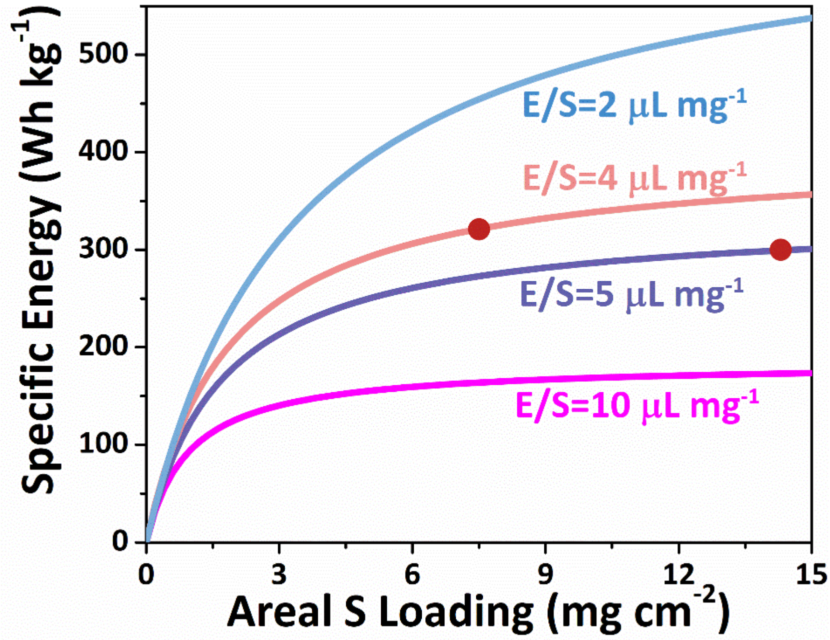

Supplementary Figure 24 | Calculated specific energy versus areal S loading for Li-S pouch cells with different E/S ratios that consider all the weight of the cell.

To projection of cell specific energy (E_g) was based on the basis of the equation (4):

$$E_g = \frac{\mathrm{V} \times \mathrm{C}}{\sum m_i} \tag{4}$$

where V is the cell discharge voltage (V), C is the cell discharge capacity (mAh), and $\sum m_i$ is the total weight of the cell component, including the weight of cathode composite, Li metal anode (100% Li excess was assumed), Al (ρ_{Al}=2.70 g cm^{-3}) & Cu (ρ_{Cu}=8.96 g cm^{-3}) current collector, electrolyte, and separator ($\rho_{separator}$=0.95 g cm^{-3}). Meanwhile, during projection, an average discharge voltage of 2.05 V with the specific capacity of 1000 mAh g^{-1} were assumed. In the case of areal S loading=10 mg cm^{-2} with the areal capacity > 10 mAh cm^{-2} and the E/S=5 μL mg^{-1} the pouch cell specific energy projection is projected to be 285.7 Wh kg^{-1}.

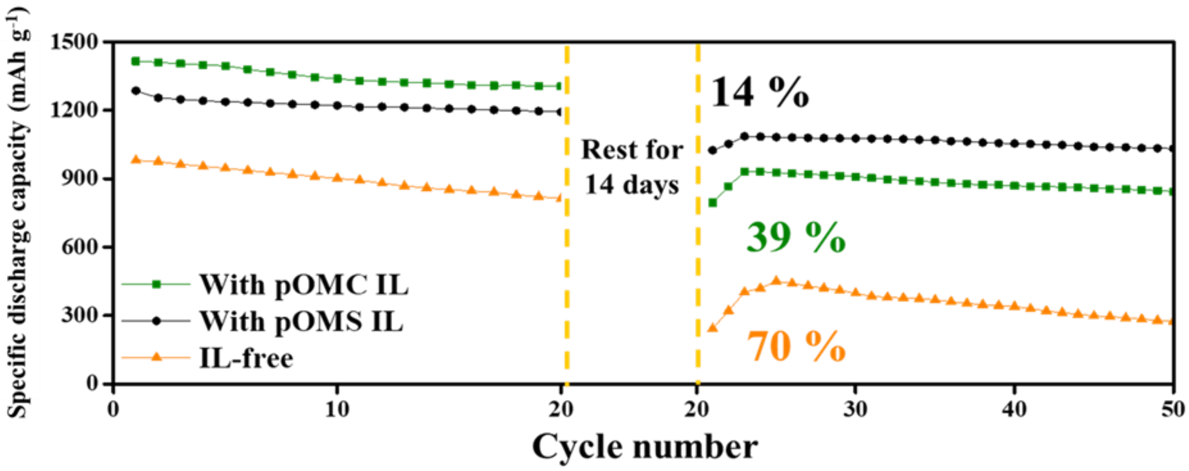

Supplementary Figure 25 | Self-discharge behaviors of pOMC IL, pOMS IL, and IL-free cells at 502.5 mA g^{-1} after rest time of 14 days at 45 °C under an E/S ratio of 10 µl mg^{-1}.

Li-S batteries suffer from self-discharge issues due to the shuttle of LiPSs and their reactions with the lithium anode, resulting in short cycle life. Working temperatures are also an important challenge in Li-S batteries because the dissolution of LiPSs into the electrolyte are accelerated at high temperature, leading to poor cycle stability[6-8]. To verify the effect of IL on the self-discharge at high temperature, the self-discharge behaviors of test cells were examined after 20 cycles at a specific current of about 502.5 mA g^{-1} in Supplementary Figure 25. The cells were rested for 14 days at 45 °C and tested for 30 additional cycles. After the rest time for 14 days at 45 °C, the IL-free cell exhibits a rapid capacity loss of 70 %, while the cells with pOMC and pOMS ILs show capacity loss of only 39 and 14 %, respectively. The low self-discharge phenomenon is attributed to the LiPSs diffusion barrier by pOMS and pOMC ILs. However, the cell with conductive pOMC IL shows a faster capacity loss compared to the one with polar pOMS IL because of weaker interaction between nonpolar pOMC IL and polar LiPSs. Thus, pOMS IL can efficiently alleviate the self-discharge behavior of Li-S batteries.

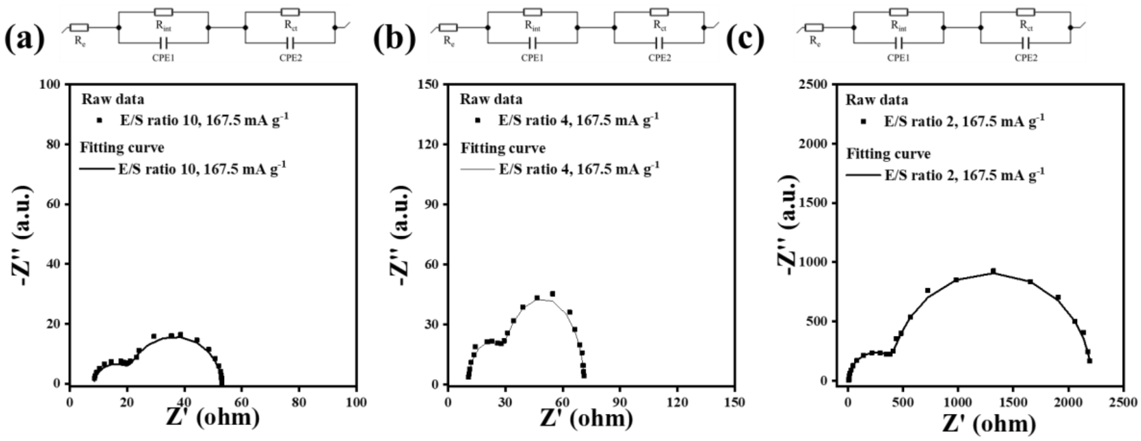

Supplementary Figure 26 | The electrochemical impedance spectroscopy measurements of the pOMS/S_{70} cathode with pOMS/S_{70} IL at E/S ratios of (a) 10 μL mg^{-1}, (b) 4 μL mg^{-1}, and (c) 2 μL mg^{-1} after 20 cycles at 167.5 mA g^{-1} and 25 ^{o}C in the fully discharge state.

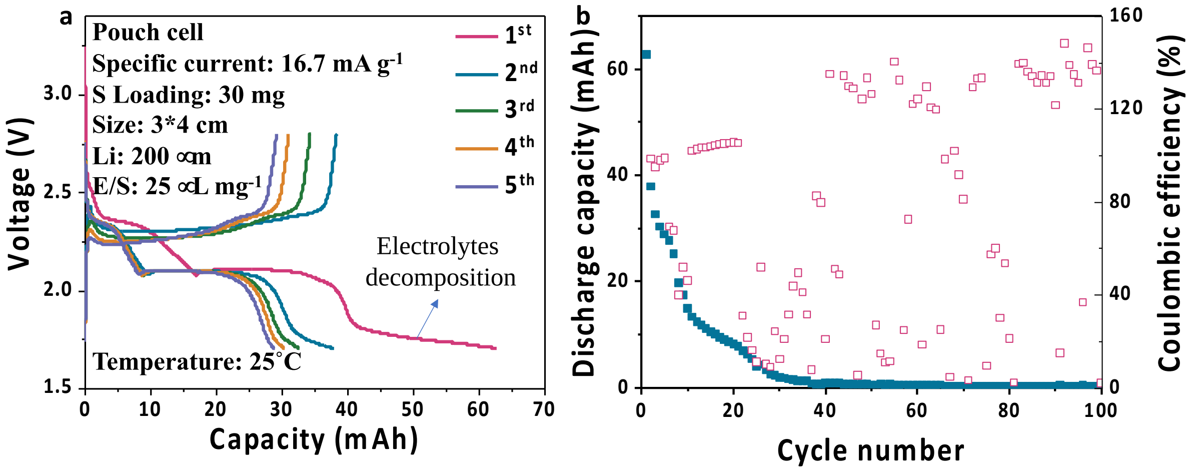

Supplementary Figure 27 | (a) Charge/discharge curve and (b) cycle performance (discharge capacity) of Li-S pouch cell using bare sulfur cathode (The pure sulfur cathode was composed of sublimed sulfur 70 wt. %, binder 10 wt. %, and electron-conducting agent 20 wt. %) and pOMS interlayer at 167.5 mA g^{-1} and 25 ^{o}C.

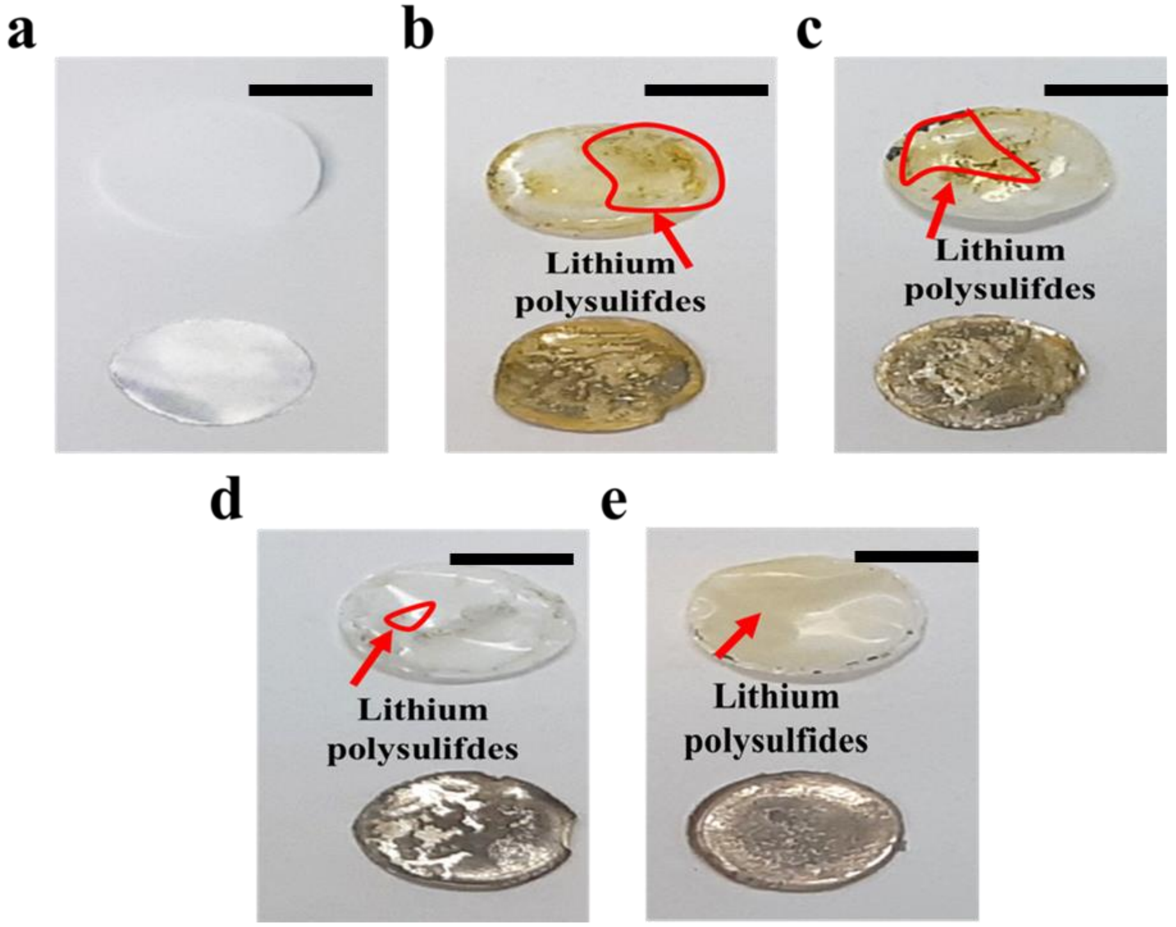

Supplementary Figure 28 | (a) Photographic pictures of the pristine separator (upper) and Li anode (lower). Photographs of separators and Li anodes obtained from the disassembled cells with (b) IL-free, (c) pOMC IL, (d) pOMS IL, and (e) pOMS/S_{50} IL after 100 cycles at 837.5 mA g^{-1} and 25 ^{o}C under an E/S ratio of 10 µl mg^{-1} (Scale bar is 10 mm).

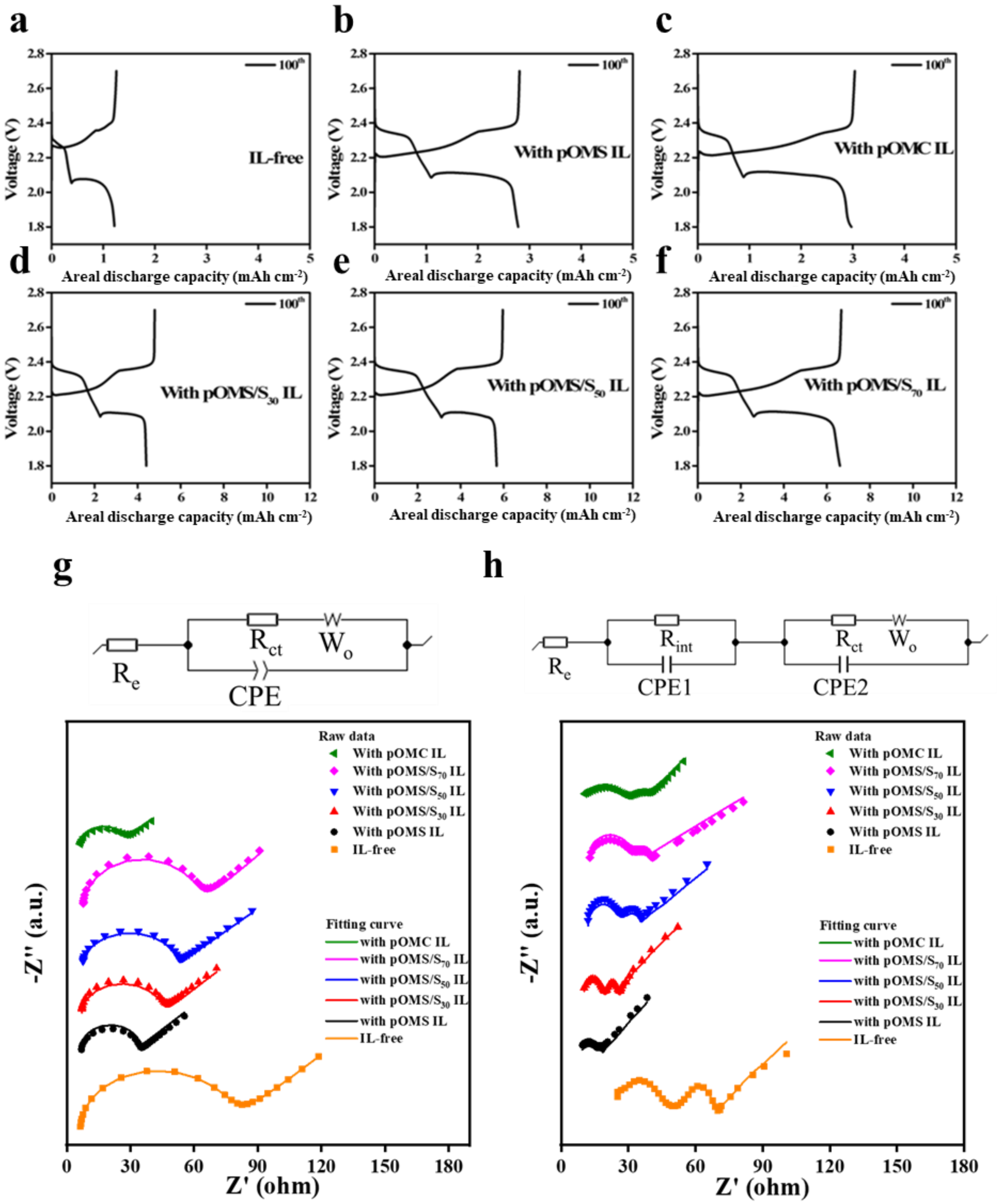

Supplementary Figure 29 | (a-f) Galvanostatic charge-discharge profiles for cells with IL-free and different ILs at a charge/discharge rate of 837.5 mA g^{-1} at 100 cycles. EIS plots of Li-S batteries employing different IL (g) before and (h) after 100 cycles at 837.5 mA g^{-1} and 25 °C under an E/S ratio of 10 µl mg^{-1} in the fully charge state.

To further understand the internal resistance of the cells with IL-free and different ILs, electrochemical impedance spectroscopy (EIS) plots after 100 cycles of charge/discharge in the fully charged state (Supplementary Figures 29a-29f) were recorded. The Nyquist plots

were analyzed with an equivalent circuit, as listed in Supplementary Table 8. The intersection with the real axis in the high-frequency region corresponds to the electrolyte resistance (R_e), and the semicircle in the high-frequency region represents the charge transfer resistance (R_{ct}) and capacitance (CPE) at the electrode-electrolyte interface. The slope is related to the Warburg impedance (W_0) assessed by the diffusion of Li ion. The CPE is a constant phase element representing the relative double-layer non-ideal capacitance[9,10]. The fresh cell shows a depressed semicircle in the high-to-medium frequency region. The pOMS and pOMC ILs cells show a smaller charge transfer resistance than the IL-free cell due to electrical conductivity being compensated by the conducting agent in IL or conductive pOMC framework. In addition, the cells with different sulfur loadings in the pOMS/S IL show relatively higher electrolyte and charge transfer resistances compared to the pOMS IL and pOMC IL cells due to the high electrolyte viscosity and low electrical conductivity associated with the high sulfur content in both the cathode and IL. However, even the cells with different sulfur loading in pOMS/S IL reveal much smaller charge transfer resistances than the IL-free cell because of the presence of a conducting agent in the IL (Supplementary Figure 29g). After 100 cycles at a charge/discharge specific current of 837.5 mA g^{-1} in the fully charged state, all of the cells show two depressed semicircles in the middle-to-high frequency region, followed by inclined lines at the low frequency region. The first semicircle at the high frequency region is caused by the formation of the insulating layer (Li_2S/Li_2S_2) on the electrode as the internal resistance (R_{int}) and CPE1, and the second semicircle at the middle frequency region is attributed to the charge transfer resistance (R_{ct}) and CPE2[11]. The charge transfer resistance for all cells decreases greatly owing to the re-arrangement of active sulfur material in the cathode and IL during cycling. The pOMS IL cell has significantly lower internal and charge transfer resistances than those of the IL-free and pOMC IL cells, indicating that polar pOMS IL prevents the dissolution and diffusion of LiPSs and thus leads to superior long-term stability (Supplementary Figure 29h). Moreover, the pOMS/S ILs cells (i.e., pOMS/S_{30}, pOMS/S_{50}, and pOMS/S_{70} ILs) have less internal resistance compared to the pOMC IL cell, indicating that polar pOMS IL can effectively inhibit LiPSs diffusion.

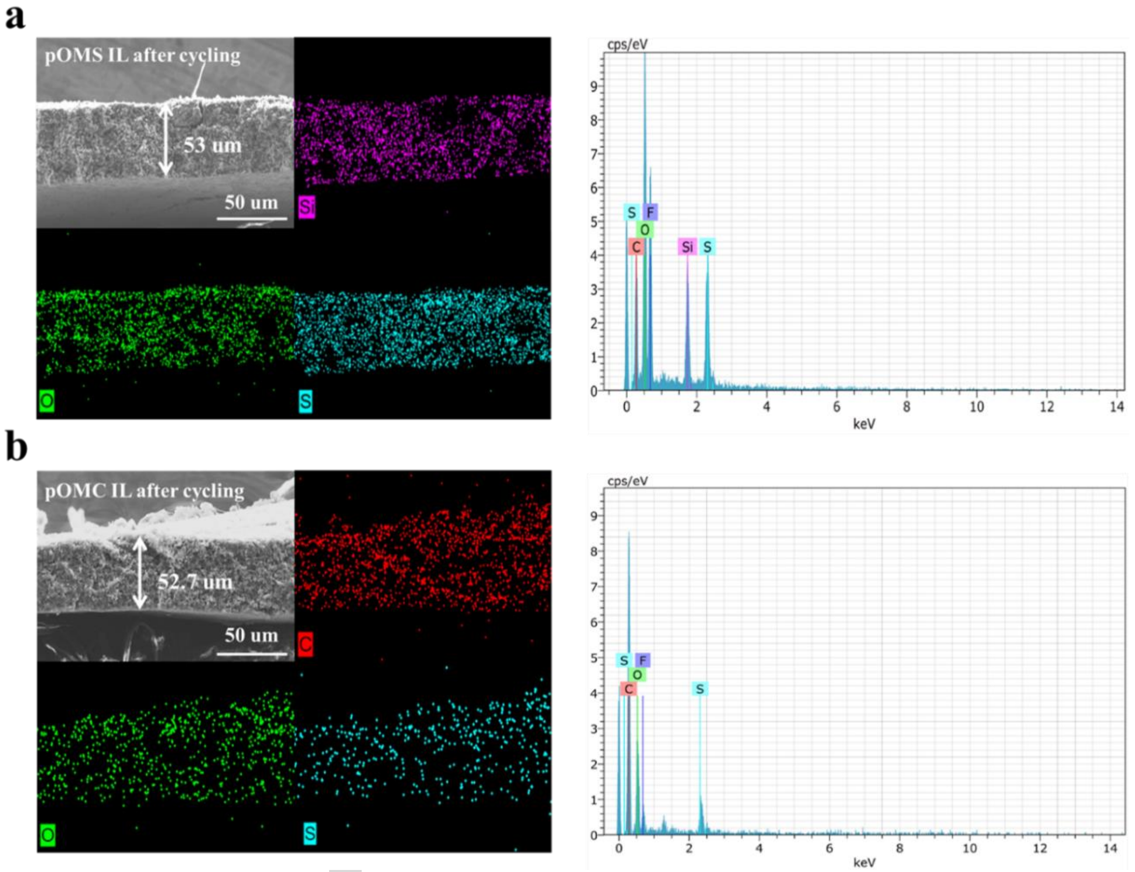

Supplementary Figure 30 | Ex situ Cross-section SEM image, EDS mappings, and corresponding EDS spectra of (a) pOMS and (b) pOMC IL after 500 cycles at 1,675 mA g^{-1} and 25 °C under an E/S ratio of 10 μl mg^{-1} in the fully discharge state.

After 500 cycles at 1675 mA g^{-1}, pOMS IL has a slight increase in thickness from 50 to 53 μm (Supplementary Figure 30a) while pOMC IL also from 50 to 52.7 μm (Supplementary Figure 30b). The corresponded elemental mappings of silicon, carbon, oxygen, and sulfur clearly illustrate their homogeneous distribution in the pOMS and pOMC ILs. Interestingly, the pOMC IL shows much weaker sulfur EDS mapping intensity compared to that of the pOMS IL as shown in Supplementary Figures 30a and 30b. The oxygen EDS mapping signal in the pOMC IL is obtained from the LiTFSI electrolyte salt or exposure to air during sample transfer. This indicates the weaker confinement of sulfur in the pOMC IL because the interaction between the non-polar pOMC IL and polar LiPSs is not sufficient to strongly trap the LiPSs during cycling, leading to rapid capacity decay over long-term cycling. On the other hand, LiPSs are strongly immobilized in the pOMS IL during the electrochemical reaction due to stronger interaction between polar pOMS IL and polar LiPSs.

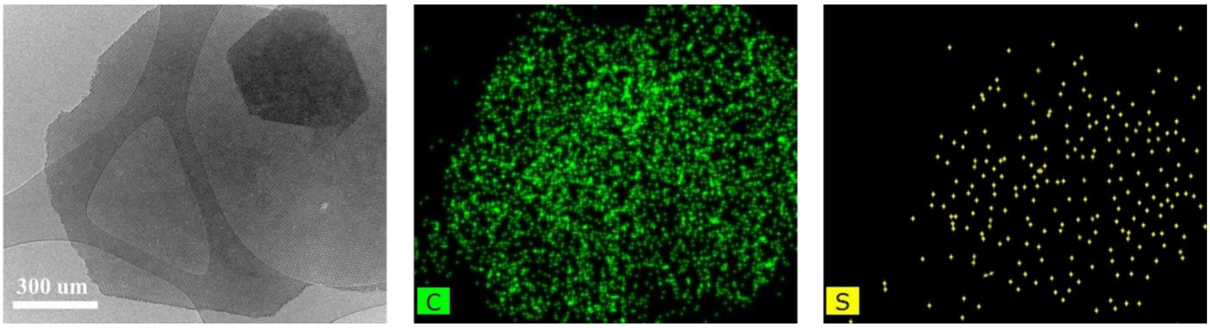

Supplementary Figure 31 | TEM image and elemental mapping of pOMC IL after 100 cycles at 837.5 mA g^{-1} and 25 ^{o}C under an E/S ratio of 10 µl mg^{-1} in the fully discharge state.

TEM characterization on the cycled pOMC IL shows that the morphology is well maintained during cycling. However, the sulfur mapping is weak compared to cycled pOMS IL because pOMC IL with a non-polar nature has weak affinity for polar LiPSs.

Supplementary Information Table 1. Analysis of the various strategies reported in the literature to improve Li-S battery performances (Li metal is always used as the negative electrode and 1 C is corresponding to ≈ 1,675 mA g^{-1})

Material	Strategy	Sulfur loading (mg cm^{-2})	1st/last areal discharge capacity (mAh cm^{-2})	E/S ratio (μl mg^{-1})	Cycle/ C-rate	Capacity fading rate (%)	Reference
Amino functional group polymer	Binder	8.0	7.9/6.6	10	50/ 0.1 C	0.329	12
PEB-1	Binder	8.1	8.13/5.42	10	100 /0.2 C	0.33	13
Ni-coated melamine foam	Current collector	8	5.66/4.7	N/A	100/ 0.5 C	0.17	14
rGO-VS_2	Cathode	2.56	2.6/2.31	10	100/ 0.1 C	0.11	15
Black phosphoursph osphorus	Interlayer	2	1.86/1.6	N/A	100/ 0.24 C	0.14	16
PDAT	Binder	4.1	3.2/2.1	12	100/ 0.1 C	0.34	17
BTO	Cathode additive	2.4	2.74/2	N/A	100/ 0.2 C	0.27	18
Li_xSi/graphene foil	Anode	1	1.09/0.86	N/A	110/ 0.5 C	0.19	19
TiC/C/Li	anode	3	3.15/2.67	N/A	200/ 0.5 C	0.076	20
3D porous graphitic carbon	Cathode	2.36	3.26/2.03	N/A	200/ 0.5 C	0.19	21
Alucone coating	Cathode	4	4.75/2.82	N/A	300/ 0.2 C	0.135	22
N-doping carbon dot	Electrolyte additive	2	1.77/1.18	20	500/ 0.5 C	0.067	23
MOF@GO	Interlayer	0.8	0.9/0.64	N/A	500/ 0.5 C	0.06	24
TiO@C-HS	Cathode	1.5	1.78/1.13	20	500/ 0.2 C	0.074	25
HKUST-1/CNT	Cathode	1	1.26/0.76	50	500/ 0.2 C	0.08	26
CoO NSs	Cathode	2	2.5/2.0	10	500/1 C	0.04	27
Nb_2O_5-MnO_2	Cathode	7	7/5.37	7	50/0.1 C	0.466	28
Se_xSPAN	Cathode	3	4.5/3.75	10	80/0.2 C	0.208	29
Ketjen black	Cathode	5	5.5/~3	16	100/0.1 C	N/A	30
Glucose + carbon coated IL	Binder + Interlayer	10.5	12/10	~9	40/0.1 C	0.417	31
$Ti_{0.87}O2$	Separator	8.9	~8	N/A	500/0.2 C	N/A	32
BTT	Li metal protection	4.8	5.47/2.79	6	60/0.2 C	0.817	33

MFC-Li/CNF	Li metal protection	6.8	6.8/6.4	14.7	200/0.1 C	0.029	34
LiBr	Electrolytes	3	~4.8/4.5	30	80/0.2 C	N/A	35

Supplementary Information Table 2. Analysis of the various interlayer (IL) strategies reported in the literature to improve Li-S battery performances (Li metal is always used as the negative electrode and 1 C is corresponding to ≈ 1,675 mA g^{-1})

Cathode materials	Interlayer materials	Sulfur loading (mg cm^{-2})	1st/last areal discharged capacity (mAh cm^{-2})	E/S ratio (µl mg^{-1})	Cycle/ C-rate	Capacity fading rate (%)/ IL thickness (µm)	Reference
Pure sulfur	rGO	1.1	1.49/0.8	40	100/ 0.2 C	0.46/22	36
Pure sulfur	Carbon	3.37	2.93/2.73	40	30/ 0.2 C	0.23/N/A	37
Carbon/S	GO	1.1	1.78/1.32	N/A	100/ 0.2 C	0.26/0.75	38
KB/S	Graphene	3	3.11/1.83	30	100/ 0.2 C	0.41/N/A	39
KB/S	MWCNT and rGO	1.5	~2.2/1.5	30	100/ 0.2 C	0.030/N/A	40
Pure sulfur	Graphene	3	4.04/3	35	50/ 0.18 C	0.51/20	41
Pure sulfur	Porous GO/CNT	1	1.37/0.67	23	300/ 0.2 C	0.17/30	42
Pure sulfur	Porous carbon microfibers	1	1.49/0.62	40	200/ 0.2 C	0.29/80	43
Pure sulfur	Hierarchically porous carbon	1.1	1.45/1.12	85	150/ 0.2 C	0.15/50	44
Graphene/ CNT/S	Graphene/CN T	2.46	3.3/3.04	14	100/ 0.5 C	0.08/15.2	45
CNT/S	G/CNT	9.4	6.2/4.1	15	200/ 0.1 C	0.17/3.5	46

Supplementary Information Table 3. Analysis of the various polar interlayer (IL) strategies reported in the literature to improve Li-S battery performances (Li metal is always used as the negative electrode and 1 C is corresponding to ≈ 1,675 mA g^{-1})

Cathode materials	Interlayer materials	Sulfur loading (mg cm^{-2})	1st/last areal discharged capacity (mAh cm^{-2})	E/S ratio (µl mg^{-1})	Cycle/ C-rate	Capacity fading rate (%)/ IL thickness (µm)	Reference
KB/S	CeO_2	6	4.8/2.77	20	325/ 0.2 C	0.13/15.96	47
rGO/S	UiO-66-S/Nafion	1.7	1.92/1.48	10	200/ 0.2 C	0.115/4	48
Carbon/S	Polystyrene sulfonate/HKUST-1	4.3	5.34/3.23	20	500/ 0.5 C	0.079/9.2	49
Pure sulfur	Co_9S_8	5.6	5.5/4.62	10	200/ 0.1 C	0.08/N/A	50
CMK-3	TiO_2-Mxen	7.3	N/A	15	200/ 0.2 C	0.21/4	51
Carbon/S	NbC	4	N/A	25	100/ 0.5 C	0.11/N/A	52
Pure sulfur	$BaTIO_3$	3	3.37/2.8	29	50/ 0.1 C	0.34/23	53
Pure sulfur	Amino-mesoporous silica nanoplates	4	2.83/2.5	10	100/ 1 C	0.12/10	54
KB/S	Oxidized poly(acrylonitrile-co-vinylpyrrolidone)/$SnCl_2$	3	3.09/2.1	30	100/ 0.2 C	0.32/185	55
Acetylene black/graphite/S	Poly(methyl methacrylate)	0.55	0.61/0.34	67	100/ 0.108 C	0.44/25.5	56
rGO/S	$VOPO_4$	1	1.12/0.84	32	300/ 0.2 C	0.083/2	57
KB/S	Covalent triazine-based frameworks	4	2.77/2.16	10	100/ 1 C	0.22/15	58
CMK-3/S	Polydopamine-modified polyimide	1.5	2.11/1.33	15	100/ 0.5 C	0.37/20	59
Pure sulfur	SnO_2	2	1.24/0.85	25	500/ 0.2 C	0.064/N/A	60

Supplementary Information Table 4. Specific surface area and pores size of pOMC, pOMS, and various pOMS/S composites. For all values, the error is ±3%.

Sample	BET surface area (m^2 g^{-1})	Micropore volume (cm^3 g^{-1})	Mesopore volume (cm^3 g^{-1})	Average pore size (nm)
pOMS	834	0.07	1.29	7.4
pOMS/S_{30}	451	0.02	0.86	7.2
pOMS/S_{50}	303	0.01	0.57	7.0
pOMS/S_{70}	33	0.01	0.07	6.8
pOMC	1544	1.32	1.66	4.3

Supplementary Information Table 5. The total intrusion volumes and porosities of the pOMC, pOMS, and various pOMS/S ILs determined by mercury intrusion measurements. For all values, the error is ±3%.

Sample	Thickness (μm)	Total Volume (cm^3 g^{-1})	Porosity (%)
pOMC IL	**50**	**1.52**	**76**
pOMS IL	**50**	**1.31**	**74**
pOMS/S_{30} IL	**50**	**1.02**	**71**
pOMS/S_{50} IL	**50**	**0.81**	**67**
pOMS/S_{70} IL	**50**	**0.69**	**63**

Supplementary Information Table 6. Sulfur loading and electrochemical conditions of a interlayer (IL)-free coin cell and coin cells with different ILs (1 C ≈ 1,675 mA g^{-1}).

Sample	Sulfur loading of electrode (mg cm^{-2})	Sulfur content in the cathode (wt %)	Sulfur loading of interlayer (mg cm^{-2})	Sulfur content in the interlayer (wt %)	Total sulfur loading (mg cm^{-2})	Sulfur amount (mg)	E/S ratio (μl /mg)	Electrolytes amount (μl)	C-rate /cycle
**Sulfur electrode with IL-free**	**2.5**	**70**	**-**	**-**	**2.5**	3.85	10	38.5	0.2C /700
**Sulfur electrode with pOMS IL**	**2.5**	**70**	**-**	**-**	**2.5**	3.85	10	38.5	0.2C /700
**Sulfur electrode with pOMC IL**	**2.5**	**70**	**-**	**-**	**2.5**	3.85	10	38.5	0.2C /700
**Sulfur electrode with pOMS/S_{30} IL**	**2.5**	**70**	**2.0**	**24**	**4.5**	7.83	10	78.3	0.2C /700
**Sulfur electrode with pOMS/S_{50} IL**	**2.5**	**70**	**5.0**	**40**	**7.5**	13.81	10	138.1	0.2C /700
							4	55.2	0.1C /50
**Sulfur electrode with pOMS/S_{70} IL**	**2.5**	**70**	**7.5**	**56**	**10**	18.80	10	188.8	0.2C /700
**Sulfur electrode with pOMS/S_{70} IL**	**6.6**	**70**	**7.5**	**56**	**14.1**	25.03	4	100.12	0.1C /50
**pOMS/S_{70} electrode with pOMS/S_{70} IL**	**6.8**	**42**	**7.5**	**56**	**14.3**	28.52	4	114.1	0.1C /50

Supplementary Information Table 7. Comparison of the electrochemical performance in this work with Li-S battery coin cells using other various interlayers (1 C ≈ 1,675 mA g^{-1}).

Cathode materials	Interlayer materials	Anode (thickness)	Electrolyte	Sulfur loading (mg cm^{-2})	1st/last areal discharge capacity (mAh cm^{-2})	E/S ratio (μl mg^{-1})	Cycle/ C-rate	Capacity fading rate (%)/ IL thickness (μm)	Reference
CNT/S	$Ni_3(HITP)_2$	Li foil	1 M LiTFSI + DME/DOL with 2 wt % $LiNO_3$	8 (cathode)	8.44/7.24	20	200/ 0.5 C	0.07/0.97	61
CNT/S	Sb_2Se_{3-x}/rGO	Li	1 M LiTFSI + DME/DOL with 1 wt % $LiNO_3$	8.1 (cathode)	7.46/6.66	10	100/ 0.1 C	0.104/32	62
MWCNT/S	$CoSe_2$/G	Li foil	1 M LiTFSI + DME/DOL with 2 wt % $LiNO_3$	4.35 (cathode)	4.78/3.62	15	100/ 0.2 C	0.24/20	63
CNT/S	supramolecular materials	Li film	1 M LiTFSI + DME/DOL with 1 wt % $LiNO_3$	4.2 (cathode)	3.78/3.37	7.3	90/ 0.1 C	0.12/4	64
CB/S	Mo_2-Mo_2N	Li metal	1 M LiTFSI + DME/DOL with 2 wt % $LiNO_3$	4 (cathode)	3.18/2.35	7	100/ 0.2 C	0.26/9	65
Pure sulfur	MoS_2/carbon microtube textile	Li foil	1 M LiTFSI + DME/DOL with 1 wt % $LiNO_3$	4.5 (cathode)	3.42/3.17	16	80/ 0.5 C	0.09/N/A	66
MWCNT/S	Co-N-C	Li metal (0.5 mm)	1 M LiTFSI + DME/DOL with 1 wt % $LiNO_3$	3.6 (cathode)	4.1/3.03	8.8	100/ 0.1 C	0.26/N/A	67
CNT/S	Co-N_x@N-doped carbon/graphene	Li metal	1 M LiTFSI + DME/DOL with 1 wt % $LiNO_3$	10.5 (cathode)	12.5/9.7	10	100/ 0.1 C	0.22/41.3	68
Super P/S	Co/SiO_2/N-doped CNT	Li foil	1 M LiTFSI + DME/DOL with 1 wt % $LiNO_3$	5.76 (cathode)	5.37/4.67	10	35/ 0.1 C	0.37/11.3	69
Graphene/S	N-doped hierarchical graphene	Li	1 M LiTFSI + DME/DOL with 5 wt % $LiNO_3$	7.2 (cathode)	5.8/4.88	7.5	30/ 0.1 C	0.53/2.2	70
MWCNT/S	Porous carbon/Sn_4P_3	Li	1 M LiTFSI + DME/DOL with 0.2 M $LiNO_3$	5.1 (cathode)	5.6/4.42	15	30/ 0.1 C	0.7/4	71
CNT/S	NiFe layered double hydroxide/ N-doped graphene	Li foil	1 M LiTFSI + DME/DOL with 1 wt % $LiNO_3$	4.3 (cathode)	4.6/3.4	10.5	100/ 0.5 C	0.26/1.5	72
Pure sulfur	Co_7Fe_3@porous graphite carbon-CNT	Li foil	1 M LiTFSI + DME/DOL with 2 wt % $LiNO_3$	6.7 (cathode)	6.1/4.1	5	90/ 0.1 C	0.36/15	73
Sublimed sulfur	pOMS	Li metal (0.25 mm)	1 M LiTFSI + DME/DOL with 0.2 M $LiNO_3$	2.5 (cathode)	3.3/2.6	10	700/ 0.2C	0.03/50	**This work**
	pOMC			2.5 (cathode)	3.8/2.2			0.06/50	
	pOMS/S_{30}			4.5 (cathode + IL)	5.4/3.8			0.042/50	
	pOMS/S_{50}			7.5 (cathode + IL)	7.2/4.9			0.046/50	

	pOMS/S_{70}			10 (cathode + IL)	8.5/5.4			0.052/50	
	pOMS/S_{50}			7.5 (cathode + IL)	5.6/4.76	4	50/ 0.1 C	0.303/50	
pOMS/S_{70}	pOMS/S_{70}			14.3 (cathode + IL)	11.29 (2nd)/10.09	5	50/ 0.1 C	0.21/50	

Supplementary Information Table 8. Calculated R_e, R_{int}, R_{ct}, and errors by fitting the EIS data (Supplementary Figure 26) for different cells before and after cycling (0.1 C = 167.5 mA g^{-1}).

**Sample**		**pOMS/S_{70} cathode with pOMS/S_{70} IL**					
		**10** μL mg^{-1}		**4** μL mg^{-1}		**2** μL mg^{-1}	
		value (ohm)	error (%)	value (ohm)	error (%)	value (ohm)	error (%)
**After 20 cycles at 0.1 C**	**R_e**	8.5	1.62	10.7	1.97	12.4	2.01
	**R_{int}**	14.7	3.45	20.5	4.62	424.6	4.28
	**R_{ct}**	23.6	4.71	41.8	6.33	1756.6	8.72

Supplementary Information Table 9. Calculated R_e, R_{int}, R_{ct}, and errors by fitting the EIS data (Supplementary Figure 29g and 29h) for different cells before and after cycling (0.5 C = 837.5 mA g^{-1}).

Sample		Pure sulfur/IL-free		pOMS IL		pOMC IL		$pOMS/S_{30}$ IL		$pOMS/S_{50}$ IL		$pOMS/S_{70}$ IL	
		value (ohm)	error (%)	value (ohm)	error (%)	value (ohm)	error (%)	value (ohm)	error (%)	value (ohm)	error (%)	value (ohm)	error (%)
**Before Cycling**	**R_e**	6.5	1.84	6.2	2.76	6.3	1.28	7.0	1.97	7.6	2.35	7.7	1.37
	**R_{int}**	-	-	-	-	-	-	-	-	-	-	-	-
	**R_{ct}**	68.9	2.20	29.1	2.82	21.4	1.39	33.2	2.93	41.5	1.48	56.9	2.76
**After 100 cycles at 0.5 C**	**R_e**	25.2	2.98	9.4	3.81	10.3	3.39	10.2	3.71	11.9	1.78	12.7	2.48
	**R_{int}**	24.3	3.11	5.9	3.79	19.1	2.13	9.3	3.64	15.0	2.54	18.6	2.59
	**R_{ct}**	20	1.45	3.2	3.41	8.1	1.42	6.6	3.51	9.1	1.95	9.3	1.63

Supplementary References

1. Lee, B.-J. et al., Revisiting the role of conductivity and polarity of host materials for long-life lithium–sulfur battery. *Adv. Energy Mater.* **10**, 1903934 (2020).
2. Yang, D. S., Bhattacharjya, D., Inamdar, S., Park, J. & Yu, J.-S. Phosphorus-doped ordered mesoporous carbons with different lengths as efficient metal-free electrocatalysts for oxygen reduction reaction in alkaline media. *J. Am. Chem. Soc.* **134**, 16127-16130 (2012).
3. Xu, M. et al., Aspergillus flavus Conidia-derived Carbon/Sulfur Composite as a Cathode Material for High Performance Lithium-Sulfur Battery. *Sci. Rep.* **6**, 18739 (2016).
4. Xu, K. et al., Manipulating the redox kinetics of Li–S chemistry by tellurium doping for improved Li–S batteries. *ACS Energy Lett.* **3**, 420-427 (2018).
5. Zhang, J. et al., Microemulsion assisted assembly of 3D porous S/graphene@g-C_3N_4 hybrid sponge as free-standing cathodes for high energy density Li-S batteries. *Adv. Energy Mater.* **8**, 1702839 (2018).
6. Zhou, Z. et al., A Multifunctional separator enables safe and durable lithium/magnesium–sulfur batteries under elevated temperature. *Adv. Energy Mater.* **10**, 1902023 (2019).
7. Li, X. et al., Safe and Durable High-Temperature Lithium-Sulfur Batteries via Moleculer Layer Deposited Coating. *Nano Lett.* **16**, 3545-3549 (2016).
8. Chen, T., et al., Self-Templated Formation of Interlaced Carbon Nanotubes Threaded Hollow Co_3S_4 Nanoboxes for High-Rate and Heat-Resistant Lithium-Sulfur Batteries. *J. Am. Chem. Soc.* **139**, 12710-12715 (2017).
9. Xiao, K. et al., Improving Polysulfides Adsorption and Redox Kinetics by the Co_4N Nanoparticle/N-Doped Carbon Composites for Lithium-Sulfur Batteries. *Small* **15**, 1901454 (2019).
10. Liu, T. et al., A robust and low-cost biomass carbon fiber@SiO_2 interlayer for reliable lithium-sulfur batteries. *Electrochim. Acta* **295**, 684-692 (2019).
11. Pu, J. et al., Multifunctional Co_3S_4@sulfur nanotubes for enhanced lithium-sulfur battery performance. *Nano Energy* **37**, 7-14 (2017).
12. Chen, W. et al., A new type of multifunctional polar binder: Toward practical application of high energy lithium sulfur batteries. *Adv. Mater.* **29**, (2017).

13. Li, L. et al., Molecular understanding of polyelectrolyte binders that actively regulate ion transport in sulfur cathodes. *Nat. Commun.* **8**, 2277 (2017).

14. Zhou, G. et al., Supercooled liquid sulfur maintained in three-dimensional current collector for high-performance Li-S batteries. *Sci. Adv.* **6**, 5098 (2020).

15. Cheng, Z., Xiao, Z., Pan, H., Wang, S. & Wang, R. Elastic sandwich-type rGO-VS_2/S composites with high tap density: Structural and chemical cooperativity enabling lithium-sulfur batteries with high energy density. *Adv. Energy Mater.* **8**, 1702337 (2018).

16. Sun, J. et al., Entrapment of polysulfides by a black-phosphorus-modified separator for lithium-sulfur batteries. *Adv. Mater.* **28**, 9797-9803 (2016).

17. Su, H. et al., Polycation binders: An effective approach toward lithium polysulfide sequestration in Li–S batteries. *ACS Energy Lett.* **2**, 2591-2597 (2017).

18. Xie, K. et al., Ferroelectric-enhanced polysulfide trapping for lithium-sulfur battery improvement. *Adv. Mater.* **29**, 1604724 (2017).

19. Zhao, J. et al., Air-stable and freestanding lithium alloy/graphene foil as an alternative to lithium metal anodes. *Nat. Nanotechnol.* **12**, 993-999 (2017).

20. Liu, S. et al., 3D TiC/C core/shell nanowire skeleton for dendrite-free and long-life lithium metal anode. *Adv. Energy Mater.* **8**, 1702322 (2018).

21. Li, G. et al., Three-dimensional porous carbon composites containing high sulfur nanoparticle content for high-performance lithium-sulfur batteries. *Nat. Commun.* **7**, 10601 (2016).

22. Li, X. et al., A high-energy sulfur cathode in carbonate electrolyte by eliminating polysulfides via solid-phase lithium-sulfur transformation. *Nat. Commun.* **9**, 4509 (2018).

23. Fu, Y. et al., Switchable encapsulation of polysulfides in the transition between sulfur and lithium sulfide. *Nat. Commun.* **11**, 845 (2020).

24. Bai, S., Liu, X., Zhu, K., Wu, S. & Zhou, H. Metal–organic framework-based separator for lithium–sulfur batteries. *Nat. Energy* **1**, 16094 (2016).

25. Li, Z. et al., A sulfur host based on titanium monoxide@carbon hollow spheres for advanced lithium-sulfur batteries. *Nat. Commun.* **7**, 13065 (2016).

26. Mao, Y. et al., Foldable interpenetrated metal-organic frameworks/carbon nanotubes thin film for lithium-sulfur batteries. *Nat. Commun.* **8**, 14628 (2017).

27. Li, R., et al., Amorphization-induced surface eletronic states modulation of cobaltous oxide nanosheets for lithium-sulfur batteries. *Nat. Commun.* **12**, 3102 (2021).

28. Liu, F., et al., Dual redox mediators accelerate the eletrochemical kinetics of lithium-sulfur batteries. *Nat. Commun*. **11**, 5215 (2020).
29. Chen, X., et al., Ether-compatible sulfurized polyacrylonitrile cathode with excellent performance enabled by fast kinetics via selenium doping. *Nat. Commun*. **10**, 1021 (2019).
30. Kang, N., et al., Cathode porosity is a missing key parameter to optimize lithium-sulfur battery energy density. *Nat. Commun*. **10**, 4597 (2019).
31. Huang, Y., et al., A saccharide-based binder for efficient polysulfide regulations in Li-S batteries. *Nat. Commun*. **12**, 5375 (2021).
32. Xiong, P., et al., Atomic-scale regulation of anionic and cationic migration in alkali metal batteries. *Nat. Commun*. **12**, 4184 (2021).
33. Guo, W., et al., Artificial dual solid-eletrolyte interfaces based on in situ organothiol transformation in lithium sulfur battery. *Nat. Commun*. **12**, 3031 (2021).
34. Ren, Y. X., et al., Rational design of spontaneous reactions for protecting porous lithium eletrodes in lithium-sulfur battereis. *Nat. Commun*. **10**, 3249 (2019).
35. Chu, H., et al., Achieving three-dimensional lithium sulfide growth in lithium-sulfur batteries using high-donor-number anions. *Nat. Commun*. **10**, 188 (2019).
36. Zhu, P. et al., Effect of reduced graphene oxide reduction degree on the performance of polysulfide rejection in lithium-sulfur batteries. *Carbon* **126**, 594-600 (2018).
37. Zhu, J. et al., A novel separator coated by carbon for achieving exceptional high performance lithium-sulfur batteries. *Nano Energy* **20**, 176-184 (2016).
38. Shaibani, M. et al., Suppressed polysulfide crossover in Li-S batteries through a high-flux graphene oxide membrane supported on a sulfur cathode. *ACS Nano* **10**, 7768-7779 (2016).
39. Cengiz, E. C., Salihoglu, O., Ozturk, O., Kocabas, C. & Demir-Cakan, R. Ultra-lightweight chemical vapor deposition grown multilayered graphene coatings on paper separator as interlayer in lithium-sulfur batteries. *J. Alloys Compd.* **777**, 1017-1024 (2019).
40. Sun, X., Huang, Y., Chen, M. & Dou, W. Double interlayers to improve cycle performance for Li–S batteries by using multiwall carbon nanotubes/reduced graphene oxide. *Ind. Eng. Chem. Res.* **57**, 6741-6745 (2018).
41. Zhou, G. et al., A graphene-pure-sulfur sandwich structure for ultrafast, long-life lithium-sulfur batteries. *Adv. Mater.* **26**, 625-631, 664 (2014).

42. Huang, J.-Q., Xu, Z.-L., Abouali, S. Akbari Garakani, M. & Kim, J.-K. Porous graphene oxide/carbon nanotube hybrid films as interlayer for lithium-sulfur batteries. *Carbon* **99**, 624-632 (2016).
43. Yanilmaz, M., Asiri, A. M. & Zhang, X. Centrifugally spun porous carbon microfibers as interlayer for Li–S batteries. *J. Mater. Sci.* **55**, 3538-3548 (2019).
44. Choi, C. & Kim, D.-W. Silica-templated hierarchically porous carbon modified separators for lithium–sulfur batteries with superior cycling stabilities. *J. Power Sources* **448**, 227462 (2020).
45. Shi, H. et al., Free-standing integrated cathode derived from 3D graphene/carbon nanotube aerogels serving as binder-free sulfur host and interlayer for ultrahigh volumetric-energy-density lithium sulfur batteries. *Nano Energy* **60**, 743-751 (2019).
46. Shi, H. et al., Easy fabrication of flexible and multilayer nanocarbon-based cathodes with a high unreal sulfur loading by electrostatic spraying for lithium-sulfur batteries. *Carbon* **138**, 18-25 (2018).
47. Wu, L., Wang, Z., An, C. & He, G. Chemical-dealloying to fabricate nonconductive interlayers for high-loading lithium sulfur batteries. *J. Alloys Compd.* **806**, 881-888 (2019).
48. Kim, S. H., Yeon, J. S., Kim, R., Choi, K. M. & Park, H. S. A functional separator coated with sulfonated metal–organic framework/Nafion hybrids for Li–S batteries. *J. Mater. Chem. A* **6**, 24971-24978 (2018).
49. Guo, Y. et al., Blocking polysulfides and facilitating lithium-ion transport: Polystyrene sulfonate@HKUST-1 membrane for lithium-sulfur batteries. *ACS Appl. Mater. Interfaces* **10**, 30451-30459 (2018).
50. He, J., Chen, Y. & Manthiram, A. Vertical Co_9S_8 hollow nanowall arrays grown on a Celgard separator as a multifunctional polysulfide barrier for high-performance Li–S batteries. *Energy Environ. Sci.***11**, 2560-2568 (2018).
51. Jiao, L. et al., Capture and catalytic conversion of polysulfides by in situ built TiO_2-MXene heterostructures for lithium–sulfur batteries. *Adv. Energy Mater.* **9**, 1900219 (2019).
52. Cai, W. et al., Conductive nanocrystalline niobium carbide as high-efficiency polysulfides tamer for lithium-sulfur batteries. *Adv. Funct. Mater.* **28**, 1704865 (2018).
53. Yim, T. et al., Effective polysulfide rejection by dipole-aligned $BaTiO_3$ coated separator in lithium-sulfur batteries. *Adv. Funct. Mater.* **26**, 7817-7823 (2016).

54. Xiang, X. et al., Mesoporous silica nanoplates facilitating fast Li^+ diffusion as effective polysulfide-trapping materials for lithium–sulfur batteries. *J. Mater. Chem. A* **7**, 9110-9119 (2019).
55. Cengiz, E. C., Ozturk, O. Hayat Soytas, S. & Demir-Cakan, R. Freestanding oxidized poly(acrylonitrile-co-vinylpyrrolidone)/$SnCl_2$ nanofibers as interlayer for Lithium Sulfur batteries. *J. Power Sources* **412**, 472-479 (2019).
56. Deng, C. et al., Double-layered modified separators as shuttle suppressing interlayers for lithium-sulfur batteries. *ACS Appl. Mater. Interfaces* **11**, 541-549 (2019).
57. He, Y. et al., Developing A "polysulfide-phobic" strategy to restrain shuttle effect in lithium-sulfur batteries. *Angew. Chem. Int. Ed.* **58**, 11774-11778 (2019).
58. Shi, Q. X. et al., Large-scaled covalent triazine framework modified separator as efficient inhibit polysulfide shuttling in Li-S batteries. *Chem. Eng. J.* **375**, 121977 (2019).
59. Wang, Y., Zhang, Z., Dong, L. & Jin, Y. Reduced shuttle effect by dual synergism of lithium–sulfur batteries with polydopamine-modified polyimide separators. *J. Membr. Sci.* **595**, 117581 (2020).
60. Xiang, Y. et al., Interfacing soluble polysulfides with a SnO_2 functionalized separator: An efficient approach for improving performance of Li-S battery. *J. Membr. Sci.* **563**, 380-387 (2018).
61. Zang, Y. et al., Large-area preparation of crack-free crystalline microporous conductive membrane to upgrade high energy lithium-sulfur batteries. *Adv. Energy Mater.* **8**, 1802052 (2018).
62. Tian, Y. et al., Low-bandgap Se-deficient antimony selenide as a multifunctional polysulfide barrier toward high-performance lithium-sulfur batteries. *Adv. Mater.* **32**, e1904876 (2020).
63. Yuan, H. et al., Conductive and catalytic triple-phase interfaces enabling uniform nucleation in high-rate lithium-sulfur batteries. *Adv. Energy Mater.* **9**, 1802768 (2019).
64. Xie, J. et al., A supramolecular capsule for reversible polysulfide storage/delivery in lithium-sulfur batteries. *Angew. Chem. Int. Ed.* **56**, 16223-16227 (2017).
65. Yang, J.-L. et al., In-situ topochemical nitridation derivative MoO_2–Mo_2N binary nanobelts as multifunctional interlayer for fast-kinetic Li-sulfur batteries. *Nano Energy* **68**, 104356 (2020).

66. Yang, J. et al., Carbon microtube textile with MoS_2 nanosheets grown on both outer and inner walls as multifunctional interlayer for lithium-sulfur batteries. *Adv. Sci.* **7**, 1903260 (2020).
67. Xie, J. et al., Implanting atomic cobalt within mesoporous carbon toward highly stable lithium-sulfur batteries. *Adv. Mater.* **31**, e1903813 (2019).
68. Cheng, Z., Pan, H., Chen, J., Meng, X. & Wang, R. Separator modified by cobalt-embedded carbon nanosheets enabling chemisorption and catalytic effects of polysulfides for high-energy-density lithium-sulfur batteries. *Adv. Energy Mater.* **9**, 1901609 (2019).
69. Fang, D. et al., Spider-web-inspired nanocomposite-modified separator: Structural and chemical cooperativity inhibiting the shuttle effect in Li-S batteries. *ACS Nano* **13**, 1563-1573 (2019).
70. Li, Q. et al., Biotemplating growth of nepenthes-like N-doped graphene as a bifunctional polysulfide scavenger for Li-S batteries. *ACS Nano* **12**, 10240-10250 (2018).
71. Ye, Z. et al., Curbing polysulfide shuttling by synergistic engineering layer composed of supported Sn_4P_3 nanodots electrocatalyst in lithium-sulfur batteries. *Nano Energy* **70**, 104532 (2020).
72. Peng, H. J. et al., A cooperative interface for highly efficient lithium-sulfur batteries. *Adv. Mater.* **28**, 9551-9558 (2016).
73. Zeng, P. et al., Enhanced catalytic conversion of polysulfides using bimetallic Co_7Fe_3 for high-performance lithium-sulfur batteries. *ACS Nano* **14**, 11558-11569 (2020).
